# Hydrodynamic flow and concentration gradients in the gut enhance neutral bacterial diversity

**DOI:** 10.1073/pnas.2108671119

**Published:** 2021-12-28

**Authors:** Darka Labavić, Claude Loverdo, Anne-Florence Bitbol

**Affiliations:** ^a^CNRS, Institut de Biologie Paris-Seine, Laboratoire Jean Perrin (UMR 8237), Sorbonne Université, F-75005 Paris, France;; ^b^Institute of Bioengineering, School of Life Sciences, École Polytechnique Fédérale de Lausanne (EPFL), CH-1015 Lausanne, Switzerland;; ^c^SIB Swiss Institute of Bioinformatics, CH-1015 Lausanne, Switzerland

**Keywords:** microbial evolution, spatially structured populations, gut microbiota, hydrodynamic flow, concentration gradients

## Abstract

The gut microbiota features important genetic diversity, and the specific spatial features of the gut may shape evolution within this environment. We investigate the fixation probability of neutral bacterial mutants within a minimal model of the gut that includes hydrodynamic flow and resulting gradients of food and bacterial concentrations. We find that this fixation probability is substantially increased, compared with an equivalent well-mixed system, in the regime where the profiles of food and bacterial concentration are strongly spatially dependent. Fixation probability then becomes independent of total population size. We show that our results can be rationalized by introducing an active population, which consists of those bacteria that are actively consuming food and dividing. The active population size yields an effective population size for neutral mutant fixation probability in the gut.

## Significance

The human body harbors numerous and diverse bacteria, the vast majority of which are located in the gut. These bacteria can mutate and evolve within the gut, which is their natural environment. This can have important public health implications (e.g., when gut bacteria evolve antibiotic resistance). The gut features specific characteristics, including hydrodynamic flow and resulting gradients of food and bacterial concentrations. How do these characteristics impact the evolution and diversity of gut bacteria? We demonstrate that they can substantially increase the probability that neutral mutants reach high proportions and eventually take over the population. This is because only a fraction of gut bacteria is actively dividing. Thus, the specific environment of the gut enhances neutral bacterial diversity.

In the human body, bacteria are approximately as numerous as human cells, and about 99% of these bacteria are located in the digestive tract (1). The gut microbiota is very diverse and collectively harbors more genes than there are human genes (2). One source of this genetic diversity is evolution occurring within the gut, which is the natural environment of these bacteria. Such evolution can have important public health implications, as the gut can constitute a reservoir of antibiotic resistance both in humans and in farm animals (3). How does the environment in the gut affect the evolution of bacteria? A crucial feature of the gut is the flow of its contents along its main axis and the associated gradients of concentration of food and bacteria. Going downstream along this axis, food is first ingested; then, simple nutrients are absorbed by the body; next more complex molecules are broken down by bacteria; and eventually, what remains of the food exits the system together with many bacteria, which make up from a quarter to half of fecal mass (4). These features yield a very particular spatial structure that can impact the evolution of bacteria.

Evolutionary models that investigate population spatial structure generally consider discrete patches of population with migrations between them and the same environment in each of them (5–13). Complex spatial structures are investigated through models on graphs where each individual (14–17) or each patch of population (18–21) occupies a node of the graph. Population structure can impact the rapidity of adaption (22–28) because local competition can allow the maintenance of larger genetic diversity. In simple population structures where migration is symmetric between patches (5, 6), the fixation probability of a mutant is unaffected by population structure (7, 8), unless extinctions of patches occur (11). However, more complex population structures with asymmetric migrations can impact the fixation probabilities of beneficial and deleterious mutants (13, 14, 21). In the case of the gut, the flow can be viewed as yielding asymmetric migrations, but the system is continuous. In large-scale turbulent systems, hydrodynamic flow has been shown to strongly impact fixation probabilities and fixation times (29–31). In addition, environmental gradients (e.g., of antibiotic concentration) can strongly impact evolution (32–35). How do population structure, hydrodynamic flow, and gradients shape the evolution of bacteria in the gut microbiota?

Here, we propose a minimal model of evolution of bacteria in the gut. Because most bacteria in the human digestive tract are located in the bulk of the colon lumen (1, 36), we focus on this compartment. Since most bacteria in the digestive tract have no self-motility (37, 38), we consider that they are carried passively with the digesta. The motion of the digesta is complex, but it was shown in refs. 39 and 40 that it can be approximated as a one-dimensional flow with net velocity and effective diffusion representing mixing. Within this model of the gut that includes hydrodynamic flow and resulting gradients of food and bacterial concentrations, we ask how the fixation probability of a neutral mutant compares with that in an equivalent well-mixed chemostat. We find that the structure of the gut can increase this fixation probability, specifically in the regime where the profiles of food and bacterial concentration are strongly spatially dependent. In this regime, fixation probability becomes independent of total population size, in stark contrast with a well-mixed population, where fixation probability is inversely proportional to total population size (41, 42). We show that this behavior can be understood by introducing the notion of active population, which corresponds to the fraction of the bacterial population that is actively consuming food and dividing.

## Model and Methods

Because the majority of bacteria in the human digestive tract are in the colon (36), we focus on this compartment. Within the colon, there are marked differences between bacteria associated with mucus and bacteria in the digesta (i.e., in the bulk of the colon lumen) (36). The latter constitute the majority of bacteria in the colon. Indeed, the surface area of the large intestine, including its folds, is about 2 m^2^ (43), while the mucus layer is about 100- to 300-μm thick (44) and typically comprises a few 10^8^ bacteria per milliliter in healthy samples (45), which leads to an order of magnitude of 10^11^ bacteria associated with mucus. This number is small compared with the total colon content, which is around 10^14^ bacteria (1). Since mucus-associated bacteria constitute a small minority in the colon and since their spatial structure and migration patterns are not well characterized, we focus on the bacteria present in the bulk of the colon lumen and do not model the mucus layer. Henceforth, we refer to the colon lumen by “gut” for simplicity.

**Fig. 1.**
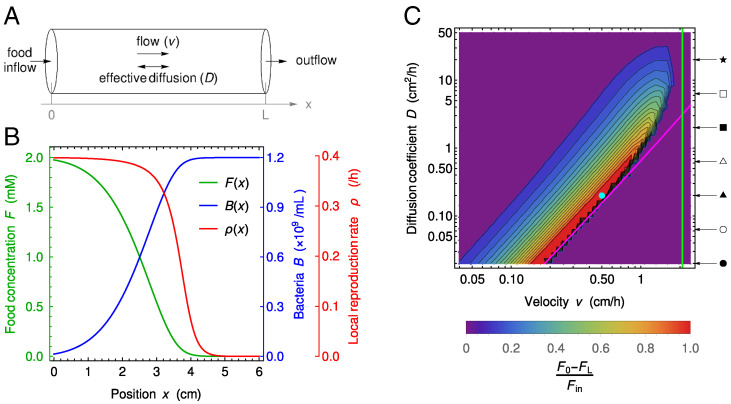
Model of the gut and associated spatial gradients. (*A*) Schematic representation of the gut model investigated. We consider a cylinder with length *L* and neglect concentration variations in the radial direction, thus simplifying the system to one dimension along the *x* axis. Transport is modeled as flow with constant velocity *v* and effective diffusion coefficient *D*. At the upstream boundary *x* = 0, we consider constant food inflow and no bacteria inflow, while at the downstream boundary *x* = *L*, we consider zero diffusive outflow. (*B*) Concentration *F* of food, amount *B* of bacteria, and reproduction rate *ρ* of bacteria vs. the coordinate *x* along the gut. Curves are numerical solutions of Eq. **1a** for D= 0.2 cm^2^/h, v= 0.5 cm/h, k= 0.1 mM, r= 0.42 h ^-1^, $v\;F_{\text{in}}=$ 1 mM cm/h, α= 6.13 × 10^8^ bacteria/mM, and no mutant bacteria. The section area, *S*, is taken to be 1 cm^2^ in the entire paper, and the length *L* is 6 cm, as in the minigut of ref. 39 in the text, but it is varied in *SI Appendix*, section S10. The parameters are chosen such that they fall in a range of parameters compared with the experiments in ref. 39 and that the concentration profile is dependent of the spatial coordinate. The depicted concentrations represent the state of the system after numerically integrating partial differential Eq. **1** for time t= 500 h, which is sufficient to reach the steady state. (*C*) Heat map of the level of spatial dependence of the concentration profiles, quantified by $[F(0)-F(L)]/F_{\text{in}}$, vs. *v* and *D*. High values of $[F(0)-F(L)]/F_{\text{in}}$ (red) mean strong gradients in the gut. Magenta and green lines represent washout limits: $D=v^{2}(k/F_{\text{in}}+1)/(4r)$ and $v=rL/(k/F_{\text{in}}+1)$, respectively. Below the magenta line and on the right side of the green line, there are no bacteria in the gut at steady state, while in the purple region on the top left-hand side, the system is well mixed, leading to an almost uniform but nonzero concentration of bacteria in the gut. Parameter values (except *v* and *D*) are the same as in *B*. The values of *v* and *D* used in *B* are indicated by a circular cyan marker. Arrows and symbols on the right-hand side of the heat map indicate the diffusion coefficient values employed in Fig. 4 with the same symbols.

The dynamics of wild-type and mutant bacteria and food in the gut is described through three concentration fields, of food *F*, wild-type bacteria *B*, and mutant bacteria *M*, based on the description of the coupled dynamics of food and bacteria (without mutants) developed in ref. 39. The gut is represented by a tube of length *L* and cross-section with surface area *S* (Fig. 1*A*). In addition to this cylindrical symmetry, we neglect radial variations and are left with a one-dimensional model along the *x* axis, specifically a segment of length *L*. We assume a constant inflow of nutrients at the entrance of this gut segment and no inflow of bacteria. At the exit of the gut, we assume that there is a free outflow of both nutrients and bacteria. The dynamics is affected by the constant flow velocity *v*, by the mixing due to different mechanisms (e.g., peristaltic movement), which is modeled by effective diffusion with diffusion coefficient *D*, and by the harvesting of the food by bacteria, which is described by a Hill-type function with Monod constant *k* and is coupled to their growth that has maximal rate *r*. This leads to the following coupled partial differential equations:

[1a] $$\frac{\partial F}{\partial t}=D\frac{\partial^{2}F}{\partial x^{2}}-v\frac{\partial F}{\partial x}-\frac{r}{\alpha }\frac{(B+M)F}{k+F},$$

[1b] $$\frac{\partial B}{\partial t}=D\frac{\partial^{2}B}{\partial x^{2}}-v\frac{\partial B}{\partial x}+r\frac{BF}{k+F},$$

[1c] $$\frac{\partial M}{\partial t}=D\frac{\partial^{2}M}{\partial x^{2}}-v\frac{\partial M}{\partial x}+r\frac{MF}{k+F},$$

with boundary conditions

[2a] $$-D\frac{\partial [F;B;M]}{\partial x}(x=0)+v[F;B;M](x=0)=[vF_{\text{in}};0;0],$$

[2b] $$-D\frac{\partial [F;B;M]}{\partial x}(x=L)=[0;0;0],$$

where [F;B;M] denotes a vector. Here, $vF_{\text{in}}$ is the food inflow at the entrance of the gut segment, while *α* denotes the yield of the conversion from food to bacteria. Note that there is zero inflow of bacteria, in agreement with observations that bacterial concentration in the smaller intestine is orders of magnitude smaller (36, 39, 40). The boundary conditions at *x* = *L* cancel the diffusive flux, corresponding to free outflow toward the downstream part of the colon.

In our study of the fate of mutants appearing in the gut, initial conditions are

[3a] $$F(t=0,x)=F^{*}(x),$$

[3b] $$B(t=0,x)=B^{*}(x),$$

[3c] $$M(t=0,x)=\{\begin{array}{l} M_{0},|x-x_{\text{M}}|\leq \Delta x/2,\\ 0,|x-x_{\text{M}}|>\Delta x/2, \end{array}$$

where $F^{*}$ and $B^{*}$ represent the steady state of system [**1a**] without mutant bacteria, while $x_{\text{M}}\in (0,L)$ is the position in the gut where the mutant appears, Δx is a short length, taken equal to the spatial discrete step in our numerical resolutions, and $M_{0}\ll B(x_{\text{M}})$ is the initial local concentration of mutant at this location. In practice, *M*_0_ is set through $N_{\text{M}}=M_{0}S\Delta x$, where *S* is the surface area of the section of the gut, so that the total number $N_{\text{M}}$ of mutants introduced in the system is always the same, and our results do not depend on Δx as long as it is small compared with the length scale over which concentrations vary.

The partial differential equations in Eq. **1** with boundary conditions in Eq. **2** and initial conditions in Eq. **3** were solved numerically (*SI Appendix*, section S1) [code is publicly available (46)].

## Results

### Spatial Dependence of the Steady-State Bacterial Concentration.

Our aim is to study the fate of neutral mutants appearing in the gut, starting from initial conditions where the concentrations of food and wild-type bacteria are at steady state (Eq. **3**). Therefore, we start by describing the steady-state profiles of food and wild-type bacteria in the mutant-free gut.

Steady-state solutions of the spatial model described by Eq. **1** can strongly depend on the spatial coordinate *x* for some values of flow velocity *v* and effective diffusion constant *D*, as exemplified by Fig. 1*B*. Such strong spatial dependence is relevant in the ascending colon (40), which is our focus here. These strongly spatial profiles resemble Fisher waves, and indeed, the steady-state equation describing our system has the same form as the equation satisfied by a traveling wave in a Fisher-Kolmogorov-Petrovsky-Piskunov (Fisher-KPP) equation (47–49). However, the velocity *v* is here an imposed parameter, in contrast to a traveling wave velocity. Moreover, the nontrivial stationary solutions satisfying the boundary conditions Eq. **2** are in a different parameter regime compared with Fisher waves (*SI Appendix*, section S3). We quantify the spatial dependence of the concentration profiles through the difference between food concentration at the entrance and at the exit of the gut, normalized by the incoming food concentration $F_{\text{in}}$, namely $[F(0)-F(L)]/F_{\text{in}}$. A heat map of this quantity is depicted in the (v,D)− parameter space in Fig. 1*C*. We observe diverse levels of spatial dependence, ranging from strongly spatial profiles to quasiflat ones, where the system is almost well mixed and resembles a chemostat, or where bacteria are washed out by the flow (39, 40) (*SI Appendix*, Fig. S1 shows examples of concentration profiles across these regimes). There are two washout limits here. First, for large diffusion coefficients, if the flow timescale is smaller than the replication timescale, bacteria exit the system before reproducing. Second, for small diffusion coefficients, on the timescale of one replication, if the characteristic length of flow is larger than that of diffusion, bacteria are washed out (*SI Appendix*, section S4 and Fig. S5).

To compare our spatial system with a well-mixed one, we consider a chemostat (50) with the same total number of bacterial reproductions $N_{\text{R}}$ per unit time as in the spatial system, which is

[4] $$N_{\text{R}}=S\int_{0}^{L}B(x)\rho (x)dx,$$

where *S* is the surface area of the gut section, while ρ(x) is the reproduction rate of bacteria, which can be expressed using food concentration as in Eq. **1**:

[5] $$\rho (x)=r\frac{F(x)}{k+F(x)}.$$

This reproduction rate strongly depends on the spatial coordinate in the spatial regime of the concentration profiles (Fig. 1*B*). After the total number of reproductions is matched, it is possible to impose an additional matching condition, and we consider three possibilities for it in *SI Appendix*, section S5. These matching conditions allow us to set the parameters characterizing the chemostat matching the spatial system, namely its dilution rate, food inflow, and volume. In all cases, we observe that matching chemostats feature extreme values for some of these parameters (*SI Appendix*, Fig. S6), which arise from the very small outflow of food in the spatial system (*SI Appendix*, section S5). These results emphasize that the large intestine is a highly efficient system for converting unabsorbed nutrients into bacteria.

### Dynamics and Fate of Neutral Mutants Appearing in the Gut.

Let us now consider neutral mutants that spontaneously appear in the gut at steady state. Mutants may appear at any position along the gut, which can feature strong spatial heterogeneities (Fig. 1). How does the initial position of these mutants affect their dynamics and their steady-state concentration?

The initial local concentration of mutants is assumed to be much smaller than that of the wild type at the position $x_{\text{M}}$ where the mutants appear (Eq. **3**), as we aim to describe the fate of a single mutant or a few mutants but in the framework of the continuous description of the gut. The early dynamics of mutant concentration is governed by the fluid dynamics in the gut. Indeed, the position *x* with the highest mutant concentration at a given time *t* initially follows the $x=x_{\text{M}}+vt$ line, while the time *t* for which the mutant concentration is maximal for a given position *x* initially follows the $t={\left(x-x_{\text{M}}\right)}^{2}/(2D)$ curves (*SI Appendix*, Fig. S7). This is consistent with the infinite space solution of the diffusion equation obtained from Eq. **1** when ignoring reproduction. Hence, transport by convective and diffusive flow allows the early spread of the mutants in the gut. Afterward, coupling with the reproduction term and the boundary conditions yields more complex dynamics.

**Fig. 2.**
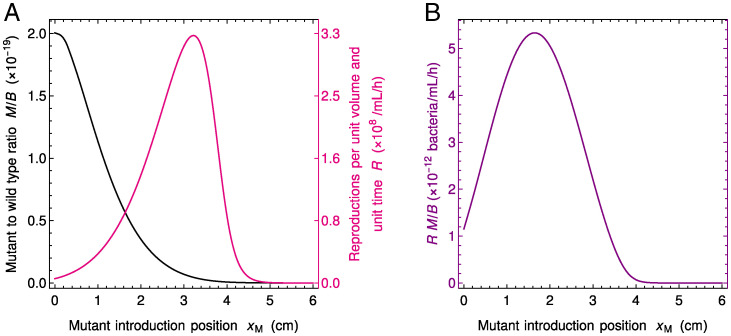
Fate of neutral mutants appearing at various locations in the gut. (*A*) Steady-state ratio *M* / *B* of mutant to wild-type bacteria concentrations and number of reproduction events *R* per unit volume and unit time vs. position $x_{\text{M}}$ of the mutant introduction. The ratio *M* / *B* yields the fixation probability of a mutant that appears at a given position $x_{\text{M}}$ in the system. As mutants generally appear upon division, the appearance of new mutants is proportional to *R*, which thus also matters for the overall likelihood that a mutant appears and fixes. (*B*) Product of the ratio *M* / *B* and the number *R* of reproductions per unit volume and unit time vs. $x_{\text{M}}$. This quantity yields the fixation probability of a mutant that appears proportionally to reproduction rate. Parameter values are the same as in Fig. 1*B*, and *F* and *B* are initially at steady state as in Fig. 1*B*, while mutants are introduced locally (in practice at numerical integration time *t* = 500 h) by using the initial condition in Eq. **3**, with a total number $N_{M}=3.33\times 10^{-11}$ of mutants introduced in the system.

Because neutral mutant concentration satisfies the same partial differential equation as wild-type bacteria concentration (Eq. **1**), the steady-state concentrations of mutant and wild-type bacteria satisfy M(x)/B(x)=C, where *C* depends on the initial conditions but not on *x*. In other words, the steady-state concentration profile of neutral mutants vs. position *x* along the gut is the same as for wild-type bacteria but with an overall rescaling. The magnitude of this rescaling (i.e., the value of *C*) depends on the initial mutant quantity and on the position $x_{\text{M}}$ where mutant bacteria appear. The latter dependence on $x_{\text{M}}$ is strong in the regime where spatial dependence is strong in the mutant-free system (Fig. 1*C*), as shown in Fig. 2*A* and *SI Appendix*, Figs. S8 and S9. If the number of mutants that appear is held constant, then mutants make up a much larger steady-state fraction of bacteria if they appeared close to the entrance of the gut than if they appeared close to its exit because they have more opportunity to spread and divide in the gut.

In our deterministic continuous description, bacterial species or strains coexist forever (except in the washout case where they are all wiped away), reflected by the fact that *M* / *B* is nonzero at steady state. However, the fate of individual mutants is in fact affected by demographic fluctuations known as genetic drift (42), so that in a finite system, mutants eventually either take over the population or disappear. Here, on a short timescale, mutants either reach deterministic steady state in coexistence with the wild type, or they get extinct stochastically. If they reach steady state, then on a longer timescale, proportional to population size (42), one of the two types takes over. What is the probability that a mutant lineage that has reached steady state then fixes in the population? In a well-mixed system, the fixation probability of a neutral mutant is given by the ratio of the number of mutants to the total number of individuals (42). In our gut model, the steady-state ratio *M* / *B* is independent of *x* in the deterministic limit [note that throughout we have M≪B so that here M/(M+B)≈M/B, and we only discuss *M* / *B* ]. Moreover, in Eq. **1**, the only nonlinearity in the evolution of *B* and *M* comes from the dependence of *F* on *B* and *M*. Here, since we introduce a very small amount of mutants, $M_{0}\ll B$, when *B* is at stationary state, and since the overall bacterial population is very large, *F* remains almost constant through the evolution of *M*, which entails that the equations for *B* and *M* are then approximately linear. Because in the linear case, the equations on averages across replicates of a stochastic system coincide with those of the deterministic large-size limit (51), the fixation probability of neutral mutants in the stochastic case is given by the deterministic steady-state ratio M/(M+B)≈M/B. In *SI Appendix*, section S12, we provide a validation of our deterministic analysis by stochastic simulations. *SI Appendix*, Fig. S16 demonstrates the good agreement between the two descriptions regarding the fate of neutral mutants appearing at various locations in the gut. In particular, it confirms that the deterministic steady-state ratio *M* / *B* yields the mutant fixation probability for each given mutant introduction position $x_{\text{M}}$. Given the dependence of the ratio *M* / *B* on the initial position $x_{\text{M}}$ of the mutants (Fig. 2*A* and discussion above), mutants appearing close to the entrance of the gut are much more likely to fix than those appearing close to its exit in the regime with strong spatial dependence (Fig. 1*C*).

Where in the gut do the mutants that fix originate? To address this question, we need to account for the apparition of mutants as well as for their fixation. Assume that mutations occur upon division, which is the case for replication errors. Then, mutants appear at a position $x_{\text{M}}$ proportionally to the local number

[6] $$R(x_{\text{M}})=B(x_{\text{M}})\rho (x_{\text{M}})$$

of reproduction events per unit volume and unit time (where the reproduction rate *ρ* is given by Eq. **5**). This number is small close to the exit of the gut because food is exhausted, but it is also small close to its entrance because bacteria are scarce, and it features a maximum at an intermediate location (Fig. 2*A*). What ultimately sets the location where mutants that fix tend to originate is the product of *M* / *B* and *R*, whose dependence on the mutant initial position $x_{\text{M}}$ is depicted in Fig. 2*B*. It features a strong spatial dependence, with a maximum at an intermediate position in the gut. In *SI Appendix*, section S8, we study *R* and R M/B for various parameter values and show that this maximum of R M/B at an intermediate position in the gut is obtained robustly in the regime with strong spatial dependence (*SI Appendix*, Fig. S10). Furthermore, *SI Appendix*, Fig. S16 demonstrates the good agreement of the results obtained in our deterministic model with those from stochastic simulations.

### Spatial Structure in the Gut Increases the Fixation Probability of Neutral Mutants.

What is the overall probability F that neutral mutants fix in the gut, averaged over their possible positions of origin? It can be expressed as the integral over all possible initial mutant locations $x_{\text{M}}$ of the fixation probability given $x_{\text{M}}$ multiplied by the probability that the mutant originates at this location $x_{\text{M}}$ :

[7] $$F=\frac{\int_{0}^{L}R(x_{\text{M}})\frac{M(x_{\text{M}})}{B(x_{\text{M}})}dx_{\text{M}}}{\int_{0}^{L}R(x_{\text{M}})dx_{\text{M}}}.$$

**Fig. 3.**
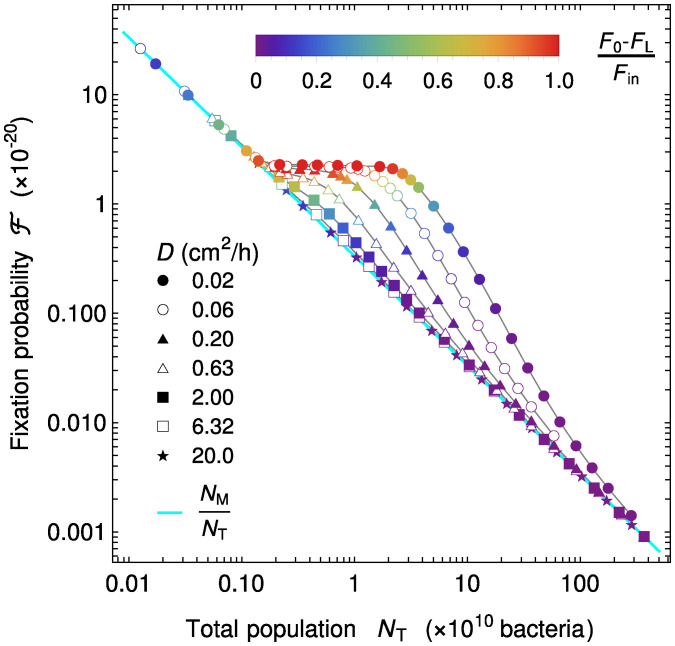
Fate of a neutral mutant vs. population size in the gut. Fixation probability F of mutants appearing proportionally to reproduction rate is shown vs. total population size $N_{\text{T}}$ for different diffusion coefficients *D* (corresponding markers are on the right-hand side of the heat map in Fig. 1*C*). Markers are colored by the level of spatial dependence of the concentration profiles, quantified by $[F(0)-F(L)]/F_{\text{in}}$ as in Fig. 1*C*. For strong spatial dependence (red), a plateau is observed, evidencing a large difference with the well-mixed expectation $F=N_{\text{M}}/N_{\text{T}}$. For each value of *D*, *v* is varied while keeping $v\;F_{\text{in}}$ constant, and fixation probability is calculated from Eq. **7** and total population by integrating the sum of the mutant and the wild type in the total space (volume). Parameter values: D∈[0.02,20.0] cm^2^/h, v∈[0.001,2.4] cm/h, k= 0.1 mM, r= 0.42 h^-1^, $v\;F_{\text{in}}=$ 1 mM cm/h, α= 6.13 × 10^8^ bacteria/mM, and initial conditions as in Fig. 2.

How is the overall fixation probability F of a neutral mutant affected by the spatial dependence of food and bacterial concentrations in the gut? To address this question, Fig. 3 depicts F vs. total population size $N_{\text{T}}=S\int_{0}^{L}B(x)dx$ for different velocities *v* and diffusion coefficients *D*. In order to include concentration profiles with different degrees of spatial dependence, quantified by $[F(0)-F(L)]/F_{\text{in}}$ (Fig. 1*C*), several values of *D* were chosen, and for each of them, a range of velocities *v* was chosen using Fig. 1*C* so that it includes flat profiles for small velocities, spatial profiles for intermediate velocities, and again, flat profiles close to the washout limit. Throughout, the food inflow $v\;F_{\text{in}}$ at the entrance of the gut was held constant to allow comparison. In a well-mixed system, we would have $F=N_{\text{M}}/N_{\text{T}}$, where $N_{\text{M}}$ denotes the initial number of mutants in the system and $N_{\text{T}}$ denotes the total number of bacteria in the system (42). We find an excellent agreement with this expectation in the case of flat concentration profiles. This is evident for small $N_{\text{T}}$ values, which correspond to the largest velocities considered and thus, to the washout limit, when the concentration profiles are the flattest (Fig. 3). Conversely, in the strongly spatial regime (red symbols in Fig. 3), the fixation probability deviates from the well-mixed system expectation, becoming substantially larger than it and almost independent of the total population. For large $N_{\text{T}}$, which corresponds to small velocities and hence, flat profiles again, the fixation probability slowly converges back to the well-mixed system expectation (Fig. 3).

### The Fixation Probability of Neutral Mutants Results from an Active Population.

**Fig. 4.**
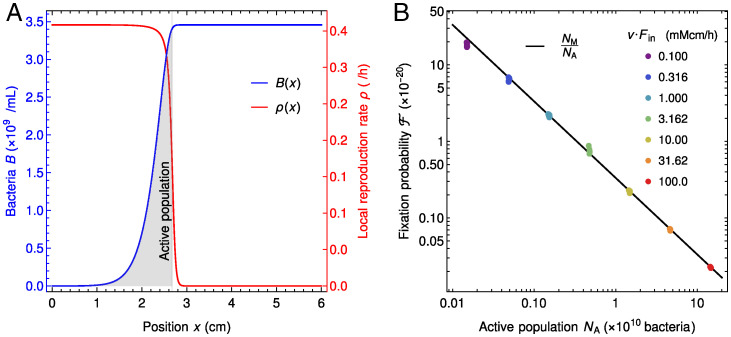
Active population explains the behavior of the neutral mutant fixation probability in the gut. (*A*) The active bacterial population (gray shaded area) is defined as the total number of bacteria between the points *x* = 0 and $x=x^{*}$, where $x^{*}$ is defined as $F(x^{*})=k$, so that $B(x^{*})=\alpha F_{\text{in}}(1-k/F_{\text{in}})$. This corresponds to the region where bacteria have significant reproduction rates. Parameters are $v\;F_{\text{in}}=$ 1 mM cm/h, v= 0.181 cm/h, D= 0.02 cm^2^/h, k=0.1 mM, r= 0.42 h^-1^, and α= 0.613 × 10^9^ bacteria/mM. (*B*) Fixation probability F of neutral mutants in the gut in the regime with strong spatial dependence vs. active population $N_{\text{A}}$ for different values of food inflow $v\;F_{\text{in}}$ (different colors). Each set of markers with a given color contains between 6 and 11 different points (often overlapping). Diffusion coefficient is the same for all points, D= 0.02 cm^2^/h; velocities are v∈[0.135,0.171] cm/h, v∈[0.14,0.18] cm/h, v∈[0.15,0.185] cm/h, v∈[0.15,0.181] cm/h, v∈[0.15,0.186] cm/h, v∈[0.155,0.182] cm/h, and v∈[0.165,0.186] cm/h for $v\;F_{\text{in}}=0.1$ to 100.0 mM cm/h, respectively. Other parameters and initial conditions are as in Fig. 3. Only the data points satisfying $\left[F(0)-F(L)\right]/F_{\text{in}}>0.9$ are retained, ensuring that we focus on the plateau of the fixation probability with respect to the total population (Fig. 3). The black line corresponds to $F=N_{\text{M}}/N_{\text{A}}$. The data shown in *A* corresponds to one of the green dots in *B*.

Why is the fixation probability of neutral mutants larger in the gut in the presence of strong spatial dependence than in a well-mixed population with the same size? An important difference is that not all bacteria are actively reproducing in the gut, while they all have the same replication rate in a well-mixed population. More precisely, in the regime with strong spatial dependence, most replications occur in the region such that the local number of reproduction events *R*(*x*) per unit volume and unit time (Eqs. **5** and **6**) is substantial (i.e., visually, under the local replication rate curve), which coincides with the zone where bacterial concentration increases (Fig. 4*A*). Quantitatively, we define the “active population” (i.e., the region with active reproduction) by comparing the replication rate with its maximum possible value (Fig. 4*A*), see *SI Appendix*, section S9 for details.

Can the active population, smaller than the total population and comprising the reproducing bacteria, quantitatively explain the fixation probability observed in the gut in the presence of strong spatial dependence? In order to assess this, we set out to significantly change active population size and thus, the total number of reproduction events, by varying the food inflow $v\;F_{\text{in}}$ at the entrance of the gut while holding the diffusion coefficient constant at D= 0.02 cm/h. We took several velocity values but only retained those such that concentration profiles were strongly spatially dependent. Fig. 4*B* shows the fixation probability F vs. the size $N_{\text{A}}$ of the active population in this spatial regime. Our results agree very well with the relation

[8] $$F=\frac{N_{\text{M}}}{N_{\text{A}}},$$

where $N_{\text{M}}$ is the initial number of mutant bacteria. This corresponds to the well-mixed expectation for the fixation probability of $N_{\text{M}}$ mutants in a population of $N_{\text{A}}$ bacteria, which confirms that the active population is the one that matters for the process of mutant fixation. This explains why the fixation probability is higher in the spatial system than in the well-mixed one, as well as the shape of the curves in Fig. 3. Indeed, in each of these curves, as *v* is decreased at a given *D*, $N_{\text{T}}$ increases. Fig. 1*C* shows that the system then goes from quasiwashout where $N_{\text{T}}$ is small and $N_{\text{A}}\approx N_{\text{T}}$ to the strongly spatial regime where $N_{\text{A}}\ll N_{\text{T}}$ and finally, toward less strongly spatial regimes where replication is slow in the whole system. When $N_{\text{A}}\approx N_{\text{T}}$, Eq. **8** reduces to the well-mixed expectation $F=N_{\text{M}}/N_{\text{T}}$, while it strongly deviates from it when $N_{\text{A}}\ll N_{\text{T}}$. Moreover, in the strongly spatial regime, almost all food is consumed. As the food inflow $vF_{\text{in}}$ is constant in Fig. 3, the overall production rate of new bacteria, which is approximately $rN_{\text{A}}$ as most reproductions occur in the active population, is then constant too, yielding a constant $N_{\text{A}}$. *SI Appendix*, Fig. S1*A* shows that when *v* is decreased within the strongly spatial regime, the transition from low to high bacterial concentration gradually shifts upstream in the gut while retaining the same shape, and thus, $N_{\text{A}}$ remains constant while $N_{\text{T}}$ increases. This explains the plateau observed in Fig. 3.

In *SI Appendix*, section S10, we demonstrate the generality of the conclusions obtained here by systematically investigating the three dimensionless parameters that fully describe the stationary state of the system. Eq. **8** holds in all cases considered, provided that the food concentration profile is strongly spatial (*SI Appendix*, Figs. S12–S14). Furthermore, we demonstrate that the range of parameters considered in the present study matches the realistic one in the human colon. Finally, *SI Appendix*, Fig. S17 shows that our prediction in Eq. **8** is validated by stochastic simulations.

## Discussion

We addressed bacterial evolution in the gut within a minimal model that incorporates flow and gradients of food and bacterial concentrations along the gut. We focused on the colon lumen, where the vast majority of our microbiota is located, and we studied parameter ranges relevant for the human colon. We considered neutral mutants appearing in the gut. Estimates of bacterial population sizes in the human colon (1, 52) and of fitness effects (53) show that a substantial fraction of spontaneous mutations occurring in gut bacteria is expected to be effectively neutral (*SI Appendix*, section S11). The dynamics of bacteria and food was described using a system of partial differential equations based on refs. 39 and 40. In the long term, in a finite-size system, mutants either disappear or take over due to stochastic fluctuations, and the stationary proportion of mutants in our continuous and deterministic description gives their fixation probability. We demonstrated that, in the regime where the profiles of food and bacterial concentrations are strongly spatial with abundant food and few bacteria upstream and vice versa downstream, the stationary concentration of mutants is higher if they start upstream. However, for mutations occurring at replication, the small upstream concentration of bacteria means that few mutants appear there. Accordingly, we found that successful mutants are more likely to originate from an intermediate position along the gut. We studied the overall long-term mutant proportion for neutral mutants appearing spontaneously upon division, which also gives their fixation probability. We found that in the almost well-mixed regime, it is given by the ratio of the initial number of mutants to the total bacterial population size, consistently with the well-mixed expectation. By contrast, when the profiles of food and bacterial concentrations are strongly spatial, which is the relevant regime in the gut (39, 40), this fixation probability becomes substantially larger than the well-mixed expectation. Thus, the spatial structure of the gut favors the spread of neutral mutants and the evolution of the population composition. Furthermore, we rationalized this increase of the fixation probability by demonstrating that it stems from the fact that only a subset of the bacterial population is actively replicating. This active population is located upstream, where there is enough food to allow substantial replication. It gives an effective population size (12, 42) for the fixation of neutral mutants in the complex structured population of the gut.

Studies addressing the impact of spatial population structure on evolution generally consider discrete patches of population with migrations between them and the same environment in each of them (5–21). While complex population structures with asymmetric migrations can impact the fixation probabilities of beneficial and deleterious mutants (13, 14, 21), that of neutral mutants appearing uniformly in the population (e.g., upon division) is unaffected (14, 21). Similarly, chaotic hydrodynamic flow has been predicted to impact nonneutral mutant fixation probabilities but not neutral ones (54). In the gut, the flow can be viewed as yielding asymmetric migrations. Strikingly, we found that the fixation probability of neutral mutants could strongly differ from the well-mixed case. Aside from the fact that the gut is a continuous system, a crucial difference with the above-cited models of population structure is that, due to directional hydrodynamic flow, the environment varies along the gut, in particular the food and bacterial concentrations and thus, the bacterial division rate. Environmental gradients can strongly impact evolution; for instance, gradients of antibiotics can increase the speed at which antibiotic resistance emerges (32–35). The coupling of bacterial concentration gradients due to antibiotics with convective flow also has complex implications on evolution (55). Hydrodynamic flow itself can strongly impact fixation probabilities and fixation times, as has been shown in the case of compressible flows relevant for large-scale turbulent systems such as bacterial populations living at the surface of oceans (29–31). In these situations, flow reduces the effective population size for fixation probability, and microorganisms born near a flow source are more likely to fix than those born in a flow sink (31). Albeit obtained in a different hydrodynamic regime, these results share similarities with ours, and together, they demonstrate that hydrodynamic flow, and in particular, convective flow, can strongly impact evolution at various scales, from the gut to the ocean.

In addition to hydrodynamic flow and gradients, the gut comprises an upstream zone with few bacteria and rapid growth. This is reminiscent of expanding fronts in populations that invade a new environment (56, 57), which feature reduced competition and reduced effective population sizes, with important consequences on evolution (58–60). In these cases, the dynamics is different depending on whether the traveling waves characterizing expansion are driven by the leading edge [pulled as, e.g., Fisher waves (47–49)] or by the bulk of the wave (pushed), yielding different wave velocities (57, 61). Contrary to population expansion on solid substrates (56), the gut features directional hydrodynamic flow. The associated velocity *v* is imposed, as opposed to a traveling wave velocity. Also, boundary conditions put us in a different parameter regime compared with Fisher waves. One may worry that our deterministic model may not be appropriate in the upstream region. However, this concern is alleviated by the directional flow, which transports bacteria downstream. Specifically, bacteria take at least 20 min to replicate (here, we took a typical replication time of 100 min), and since they are transported with the flow, the lineage of an upstream bacteria will be broadly distributed, including where there are many bacteria, before being large enough to affect *F* sufficiently to modify the dynamical equations via F/(k+F). Furthermore, the main findings from our deterministic model are validated by stochastic simulation results (*SI Appendix*, section S12).

Extending our study from neutral mutants to beneficial and deleterious ones, and studying fixation times and the rate of evolution in the gut, would be interesting topics for future work. Note that given the very large numbers of bacteria at play, fixation is expected to be slow. However, even before fixation, our results show that the proportion of mutants is increased by the gut structure compared with a well-mixed system. Our stochastic simulation results in *SI Appendix*, Fig. S18 confirm that the timescale for the increase of the average proportion of mutants is much faster than the one for mutant fixation. Furthermore, while the minimal model used here captures some key characteristics of the gut (including net flow, effective mixing, and a stable bacterial population), the reality of the gut is more complex. In particular, muscle contractions in peristalsis and segmentation (62, 63) mean that the radius of the gut is variable and yield complex mixing dynamics. Besides, several food sources and several bacterial species are present, yielding complex ecological dynamics. Bacterial populations in the colon lumen can also interact with those in the mucus and in crypts. In addition, assuming a constant food inflow is a simplification, and in real life, food inflow is variable depending, for example, on the timing of meals, thus adding time variability to the spatial gradients we considered here. Despite all these complications, our results, which can be interpreted simply through the active population, have the potential to be general and can be tested in more detailed models.

### Note.

While this manuscript was in revision, an independent and complementary study (64) was released on bioRxiv.

## Supplementary Material

Supplementary File

## Data Availability

All relevant data is included in the paper or in the *SI Appendix*. Code for simulations has been deposited in Zenodo (https://doi.org/10.5281/zenodo.4704653).
