# Supplementary material for: Hydrodynamic flow and concentration gradients in the gut enhance neutral bacterial diversity
Source: Proc Natl Acad Sci U S A. 2021 Dec 28;119(1):e2108671119. doi: 10.1073/pnas.2108671119 (PMC8740595; doi:10.1073/pnas.2108671119)
Supplement: Supplementary File [file pnas.2108671119.sapp.pdf]

Supplementary information: Hydrodynamic flow and concentration gradients in the gut enhance neutral bacterial diversity

Darka Labavić[1], Claude Loverdo[1,*,†] and Anne-Florence Bitbol[2,3,*,†]

[1]*Sorbonne Université, CNRS, Institut de Biologie Paris-Seine, Laboratoire Jean Perrin (UMR 8237), F-75005 Paris, France,*

[2]*Institute of Bioengineering, School of Life Sciences, École Polytechnique Fédérale de Lausanne (EPFL), CH-1015 Lausanne, Switzerland,*
[3]*SIB Swiss Institute of Bioinformatics, CH-1015 Lausanne, Switzerland,*
[*]*Equal contribution,*
[†]*E-mail addresses: claude.loverdo@sorbonne-universite.fr; anne-florence.bitbol@epfl.ch.*

November 22, 2021

Contents

S1 Numerical methods **2**
S1.1 Discretization of the partial differential equation system . . . 2
S1.2 Obtaining stationary profiles without and with mutants . . . 2
S1.3 Conversion between food and bacteria concentrations . . . 3

S2 Stationary profiles without mutants **3**
S2.1 Ordinary differential equation description . . . 3
S2.2 Dimensionless form . . . 4
S2.3 Some stationary profiles . . . 4

S3 Comparison to Fisher waves **6**

S4 Washout limits **8**

S5 Correspondence between the spatial system and the chemostat **11**
S5.1 Main matching condition . . . 11
S5.2 Constraints from each separate system . . . 11
S5.3 Additional matching conditions . . . 12
S5.4 Properties of the matching chemostats . . . 12

S6 Early dynamics of mutant bacteria concentration **15**

S7 Stationary state of mutant bacteria concentration versus the initial position x_{M} of mutants **16**

S8 Location where most mutants that fix originate **17**

S9 Calculation of the active population size **17**

S10 Impact of the dimensionless parameters **18**
S10.1 Holding the dimensionless parameters fixed . . . 18
S10.2 Varying each dimensionless parameter . . . 19
S10.3 Range of the dimensionless parameters and relevance for the human colon . . . 20

S11 Relevance of neutral mutations **21**

S12 Validation by stochastic simulations **22**
S12.1 Stochastic simulation methods . . . 22
S12.2 Stochastic simulation results . . . 24

S1 Numerical methods

S1.1 Discretization of the partial differential equation system

In order to solve the system 1 numerically, we employ an explicit method using a forward difference for the time derivative at time t and a central difference for the space derivative at position x. Explicitly, the relevant differential operators are replaced by the following expressions:

$$\frac{\partial Y}{\partial t} \to \frac{Y(t,x) - Y(t-\Delta t, x)}{\Delta t}, \tag{S1a}$$

$$\frac{\partial Y}{\partial x} \to \frac{Y(t,x+\Delta x) - Y(t, x-\Delta x)}{2\Delta x}, \tag{S1b}$$

$$\frac{\partial^2 Y}{\partial x^2} \to \frac{Y(t,x+\Delta x) - 2Y(t,x) + Y(t, x-\Delta x)}{(\Delta x)^2}, \tag{S1c}$$

where Δt and Δx represent the discrete steps in time and space, respectively. Here $Y(t,x)$ can represent the concentration of food F, or wild type bacteria B, or mutant bacteria M, at time t and coordinate x. Substituting the differential operators in Eqs. 1 using Eqs. S1 yields

$$\begin{aligned} F(t+\Delta t, x) = F(t,x) + D\frac{F(t,x+\Delta x) - 2F(t,x) + F(t,x-\Delta x)}{(\Delta x)^2}\Delta t - \\ - v\frac{F(t,x+\Delta x) - F(t,x-\Delta x)}{2\Delta x}\Delta t - \frac{r}{\alpha}(B(t,x) + M(t,x))\frac{F(t,x)}{k + F(t,x)}\Delta t, \end{aligned} \tag{S2a}$$

$$\begin{aligned} B(t+\Delta t, x) = B(t,x) + D\frac{B(t,x+\Delta x) - 2B(t,x) + B(t,x-\Delta x)}{(\Delta x)^2}\Delta t - \\ - v\frac{B(t,x+\Delta x) - B(t,x-\Delta x)}{2\Delta x}\Delta t + rB(t,x)\frac{F(t,x)}{k + F(t,x)}\Delta t, \end{aligned} \tag{S2b}$$

$$\begin{aligned} M(t+\Delta t, x) = M(t,x) + D\frac{M(t,x+\Delta x) - 2M(t,x) + M(t,x-\Delta x)}{(\Delta x)^2}\Delta t - \\ v\frac{M(t,x+\Delta x) - M(t,x-\Delta x)}{2\Delta x}\Delta t + rM(t,x)\frac{F(t,x)}{k + F(t,x)}\Delta t. \end{aligned} \tag{S2c}$$

The boundary conditions in $x = 0$ from Eqs. 2 become

$$F(t, 0-\Delta x) = F(t, 0+\Delta x) + \frac{2\Delta x}{D}v\left[F_{\text{in}} - F(t,0)\right], \tag{S3a}$$

$$B(t, 0-\Delta x) = B(t, 0+\Delta x) - \frac{2\Delta x}{D}vB(t,0), \tag{S3b}$$

$$M(t, 0-\Delta x) = M(t, 0+\Delta x) - \frac{2\Delta x}{D}vM(t,0), \tag{S3c}$$

while the boundary conditions in $x = L$ from Eqs. 2 become

$$F(t, L+\Delta x) = F(t, L-\Delta x), \tag{S4a}$$

$$B(t, L+\Delta x) = B(t, L-\Delta x), \tag{S4b}$$

$$M(t, L+\Delta x) = M(t, L-\Delta x). \tag{S4c}$$

The spatial discrete step Δx is in general chosen to be 0.01 cm. This value is small enough to ensure convergence for most model parameters, and to have a good spatial resolution for analysis. Note however that for some parameters, the term $[F_{\text{in}} - F(t,0)]$ in Eq. S3a can be very large which can lead to numerical instability. To compensate, the spatial step, Δx, needs to be reduced. Once Δx is chosen, the value of Δt should satisfy the stability condition $\Delta t \leq (\Delta x)^2/(2D)$ [1]. Specifically, we take $\Delta t = 0.8(\Delta x)^2/(2D)$.

S1.2 Obtaining stationary profiles without and with mutants

Numerically, we determine the unique solution for food concentration that satisfies $F(x) < F_{\text{in}}$ for all x. Thus, the steady-state profile of food concentration is independent of the initial conditions as long as they are all positive. In general, for our numerical integration, we choose initial conditions not too far to the steady state, namely $F(0,x) = F_0 \in (0, F_{\text{in}})$, $B(0,x) = \alpha[F_{\text{in}} - F(0,x)]$, and $M(0,x) \ll B(0,x)$, in order to obtain faster convergence. If there is no mutant bacteria, the steady state of the wild type bacteria concentration is also uniquely

defined through the relation $B(x) = \alpha[F_{\text{in}} - F(x)]$. However, if there are both wild type and mutant bacteria in the system, then the steady state solution is uniquely defined only for the total bacterial concentration, while individual concentrations depend on the initial conditions for wild type and mutant bacteria.

In practice, the stationary state in the absence of mutant bacteria is found using Eqs. S2 coded in the Fortran90 programming language (code available at `https://doi.org/10.5281/zenodo.4704653` [2]) with homogeneous initial conditions

$$F(0,x) = 0.9F_{\text{in}}, \tag{S5a}$$
$$B(0,x) = \alpha[F_{\text{in}} - F(0,x)] = 0.1\alpha F_{\text{in}}. \tag{S5b}$$

The time used in all numerical integrations is $t = 500\,\text{h}$, which is long enough for the system to reach the steady state for all choices of parameters considered in this paper.

To find the mutant concentration profile that is crucial to our calculation of the fixation probability, we consider the system without mutants at steady state, and we assume that mutants appear at one local position, x_{M}, in the gut segment. We again solve Eqs. S2 for time $t = 500\,\text{h}$, but now with the initial conditions

$$F(0,x) = F^*(0,x), \tag{S6a}$$
$$B(0,x) = B^*(0,x), \tag{S6b}$$
$$M(0,x) = \begin{cases} M_0, & |x - x_{\text{M}}| \leq \Delta x/2, \\ 0, & |x - x_{\text{M}}| > \Delta x/2, \end{cases} \tag{S6c}$$

where F^* and B^* represent steady state concentrations without mutant bacteria, while $M_0 \ll B(x_M)$ is the initial local mutant concentration. More precisely, denoting by N_{M} the total number of mutant bacteria introduced in the system, the quantity M_0 is the initial concentration in the segment $[x_{\text{M}} - \Delta x/2, x_{\text{M}} + \Delta x/2]$, where Δx is the spatial discrete step of our numerical resolutions. Hence, M_0 satisfies $N_{\text{M}} M_0 S \Delta x$, where N_{M} is the total number of mutants introduced in the system. Note that since we are using central difference discretization, we need to double the M_0 value on boundaries of the segment where the mutants are introduced, in order to have the same N_{M} there as in the rest of the segment. In practice, we choose the value $N_{\text{M}} = 3.33 \times 10^{-11}$ bacteria, so that for any choice of parameters used in this paper and for any initial mutant position x_{M}, the relation $M_0 \ll B(x_{\text{M}})$ is satisfied. Importantly, since the stationary concentration of mutant bacteria is proportional to M_0, all results scale with it, and we are not losing generality by fixing the value of M_0.

S1.3 Conversion between food and bacteria concentrations

The initial unit of food concentration is moles per liter, and that of bacterial concentration is optical density, OD [3], which can be converted to numbers of bacteria per volume by using the calibration curve in [3]. Specifically, the conversion factor we take is 1 OD=3.33×10^9 bacteria/mL. Then the parameter α allows to convert between food and bacteria concentrations.

Importantly, because α is just a scaling factor, a change in this value will modify the bacterial concentration quantitatively, but the spatial profile and all the other conclusions will remain identical.

S2 Stationary profiles without mutants

S2.1 Ordinary differential equation description

Without mutants, at stationary state, Eq. 1 yields:

$$0 = D\frac{\partial^2 F}{\partial x^2} - v\frac{\partial F}{\partial x} - \frac{r}{\alpha}\frac{FB}{k+F}\,, \tag{S7}$$
$$0 = D\frac{\partial^2 B}{\partial x^2} - v\frac{\partial B}{\partial x} + r\frac{FB}{k+F}\,, \tag{S8}$$

with boundary conditions in Eq. 2.

Introducing $f = \alpha F + B$, we have at stationary state

$$C = D\frac{\partial f}{\partial x} - vf\,, \tag{S9}$$

where C is a constant. The solution reads

$$f(x) = -\frac{C}{v} + C' e^{vx/D} \, , \tag{S10}$$

where C' is a constant. Applying the boundary conditions yields $C' = 0$ and $C = -v\alpha F_{\text{in}}$. Thus,

$$\alpha F(x) + B(x) = f(x) = \alpha F_{\text{in}} \, , \tag{S11}$$

and this specific linear combination of B and F is independent from x: food effectively gets converted into bacteria.

Now we can inject this into the equation on F to decouple it from B, yielding:

$$0 = D\frac{\partial^2 F}{\partial x^2} - v\frac{\partial F}{\partial x} - r\frac{F(F_{\text{in}} - F)}{k + F} \, , \tag{S12}$$

which is a second order nonlinear ordinary differential equation.

S2.2 Dimensionless form

Let us make the variable change $s = xv/D$, and let us introduce the function ϕ satisfying $\phi(s = xv/D) = F(x)/F_{\text{in}}$ for all x. We obtain

$$0 = \frac{\partial^2 \phi}{\partial s^2} - \frac{\partial \phi}{\partial s} - \lambda\frac{\phi(1 - \phi)}{\kappa + \phi} \, , \tag{S13}$$

which involves the dimensionless numbers

$$\kappa = \frac{k}{F_{\text{in}}} \, , \tag{S14}$$

and

$$\lambda = \frac{rD}{v^2} \, . \tag{S15}$$

The associated boundary conditions are:

$$\phi(s = 0) - \frac{\partial \phi}{\partial s}(s = 0) = 1 \, , \tag{S16}$$

$$\frac{\partial \phi}{\partial s}(s = \sigma) = 0 \, , \tag{S17}$$

where

$$\sigma = \frac{Lv}{D} \tag{S18}$$

is the third dimensionless number describing the system [3], and compares transport by convection to transport by diffusion. It has the same form as the Péclet number for mass transfer, but recall that D is an effective diffusion coefficient modeling gut contractions.

S2.3 Some stationary profiles

In practice, the partial differential equations in Eqs. 1 with boundary conditions in Eqs. 2 and initial conditions in Eqs. 3 were solved numerically as explained above. Examples of profiles obtained are given in Figure S1. Note that we checked that the long-term results from their direct resolution was consistent to those obtained by numerically solving the ordinary differential equation S12 giving the stationary state of the system.

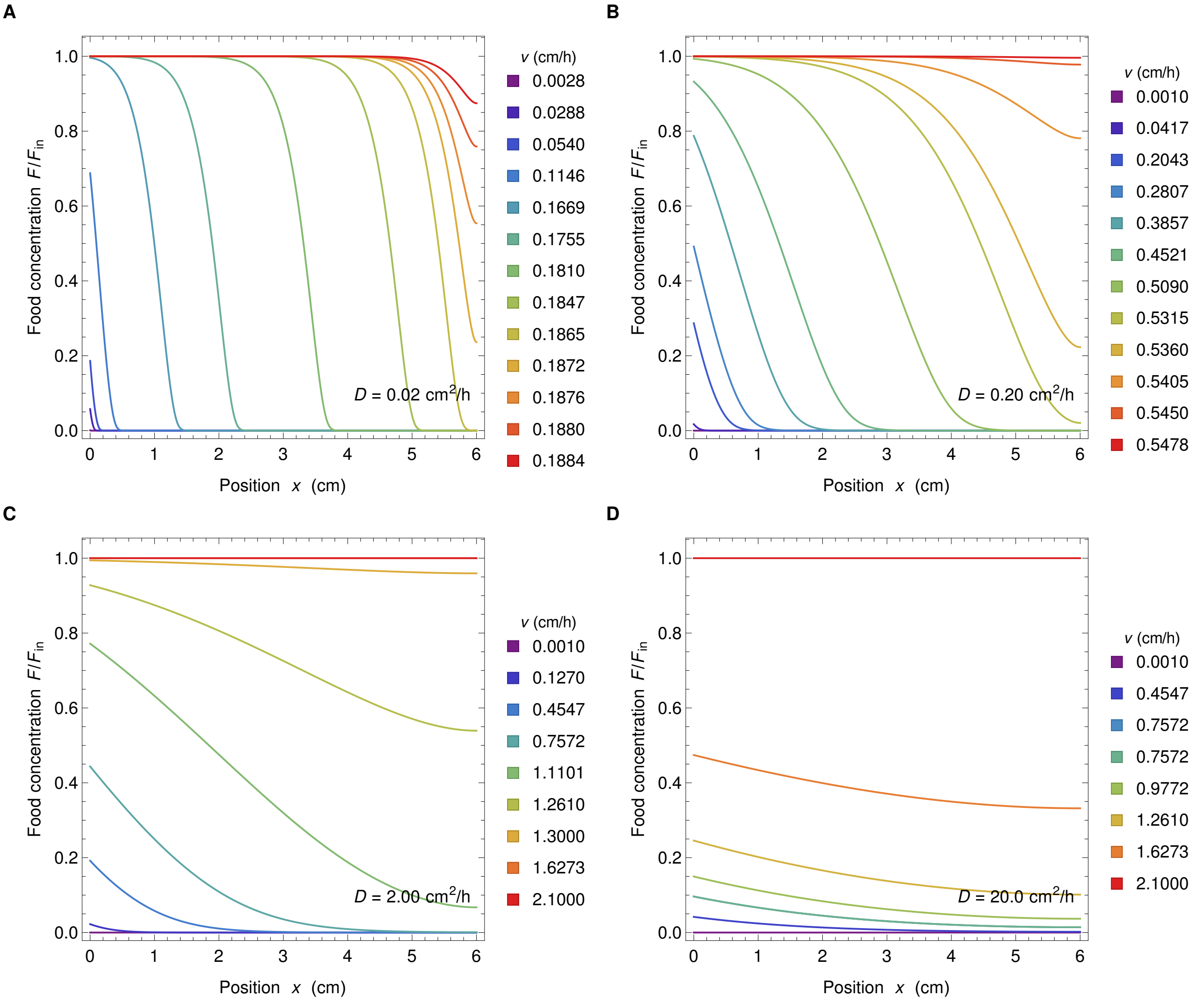

Figure S1: Food concentration profiles. Food concentration F normalized by the food concentration inflow F_{in} versus position x along the gut for four different values of the diffusion coefficient (panels A to D) and several values of velocity (different colors in each panel). Values of diffusion coefficients and velocity are indicated in each panel. Other parameter values are $k = 0.1\,\mathrm{mM}$, $r = 0.42\,\mathrm{h}^{-1}$, $v\,F_{\mathrm{in}} = 1\,\mathrm{mM\,cm/h}$, $\alpha = 6.13 \times 10^{8}$ bacteria/mM.

S3 Comparison to Fisher waves

In the regime with strong spatial dependence, the present model yields at steady state an upstream zone with few bacteria, high food concentration and rapid growth, and a downstream zone with many bacteria, little food and little growth. Specifically, our numerical resolutions show that $\phi = F/F_{\text{in}}$ then monotonically decays from a value close to one to a value close to zero, with a sigmoid-like shape (see Fig. 1B, as well as Fig. S1A-B for intermediate velocities). This is reminiscent of Fisher waves [4], which are (among several applications) an important model for the spread of mutant genes in populations [4], as well as for the expansion of populations that invade a new environment [5, 6], where the dynamics of mutants has been extensively discussed [7–13]. Let us compare these two models.

The Fisher-Kolmogorov-Petrovsky-Piskunov (Fisher-KPP) equation [4, 14] is a reaction-diffusion equation of the form [5, 15]

$$\frac{\partial u}{\partial t} = \frac{\partial^2 u}{\partial x^2} + f(u)\,, \tag{S19}$$

where f is a function satisfying $f(0) = f(1) = 0$. For $f(u) = u(1-u)$, it is known as the Fisher equation [4, 6]. Searching for a "Fisher wave" solution of Eq. S19 in the form of a traveling wave $u(x,t) = U(z = x \pm ct)$, where c is a wave velocity to be determined [5], yields:

$$\frac{d^2U}{dz^2} \pm c\frac{dU}{dz} + f(U) = 0\,. \tag{S20}$$

It is known [15] that if $f'(0) > 0$, $f'(1) < 0$, and $f(x) > 0$ for all $x \in]0,1[$, then monotonic wave front solutions such that $\lim_{z\to-\infty} U(z) = 0$ or 1, and $\lim_{z\to\infty} U(z) = 1$ or 0, exist if and only if $|c| \geq c^*$, where $c^* > 0$ is a constant that can be determined by analyzing the stability of the fixed points at $(U,U') = (0,0)$ and $(U,U') = (1,0)$. In particular, $c^* = 2$ in the case of the Fisher equation [6].

In our model, steady-state profiles of ϕ are solutions of Eq. S13, and thus, using $\xi = x/D$ instead of $s = xv/D$, the function $\psi = 1-\phi$ satisfies

$$\frac{d^2\psi}{d\xi^2} - v\frac{d\psi}{d\xi} + rD\frac{\psi(1-\psi)}{\kappa+1-\psi} = 0\,, \tag{S21}$$

which has the form of Eq. S20, with v replacing $\mp c$, and with $f : \psi \mapsto rD\,\psi(1-\psi)/(\kappa+1-\psi)$. This function satisfies $f(0) = f(1) = 0$, as well as $f'(0) > 0$, $f'(1) < 0$, and $f(x) > 0$ for all $x \in]0,1[$, since r, D and κ are positive constants. Therefore, monotonic stationary solutions such that $\lim_{z\to-\infty}\psi(z) = 0$ or 1, and $\lim_{z\to\infty}\psi(z) = 1$ or 0, exist if and only if $v \geq v^*$ (recall that in our system $v > 0$), and the same result holds for $\phi = 1-\psi$.

In order to determine v^*, let us study the stability of the fixed points at $(\phi,\phi') = (0,0)$ and $(\phi,\phi') = (1,0)$ in Eq. S13. Introducing $\chi = \phi'$, where the prime denotes a derivative with respect to $s = xv/D$, Eq. S13 becomes:

$$\begin{cases} \phi' = \chi\,, \\ \chi' = \chi + \lambda\,\phi(1-\phi)/(\kappa+\phi)\,. \end{cases} \tag{S22}$$

A linear stability analysis demonstrates that $(0,0)$ is a saddle point, while $(1,0)$ is either an unstable node if $4\lambda < \kappa+1$ (see Fig. S2A) or an unstable spiral if $4\lambda > \kappa+1$ (see Fig. S2B and C). Qualitative phase space analysis (similar to that presented e.g. in [6] for the Fisher equation) then shows that stationary solutions such that $\lim_{s\to-\infty}\phi(s) = 1$ and $\lim_{s\to\infty}\phi(s) = 0$ exist in both cases, but that they are monotonic if and only if $4\lambda \leq \kappa+1$, or equivalently

$$v \geq v^* = \sqrt{\frac{4rD}{\kappa+1}}\,. \tag{S23}$$

Therefore, in infinite space, there exists a stationary solution to the system of partial differential equations Eqs. 1 that is equivalent to a Fisher wave (in a moving frame), provided that Eq. S23 is satisfied. However, the velocity v is a parameter of the system, and not a wave velocity that can adjust, in contrast to the usual Fisher-KPP case.

The model studied in this paper employs the system of partial differential equations Eq. 1 in a finite segment $x \in [0,L]$ (or equivalently, $s \in [0, \sigma = Lv/D]$), with the boundary conditions Eq. 2, which become in dimensionless form:

$$\begin{cases} \chi(0) = \phi(0) - 1\,, \\ \chi(\sigma) = 0\,. \end{cases} \tag{S24}$$

Is there a nontrivial solution satisfying these conditions, and if yes, how does it compare to the Fisher-wave-like monotonic solution such that $\lim_{s\to-\infty}\phi(s) = 1$ and $\lim_{s\to\infty}\phi(s) = 0$?

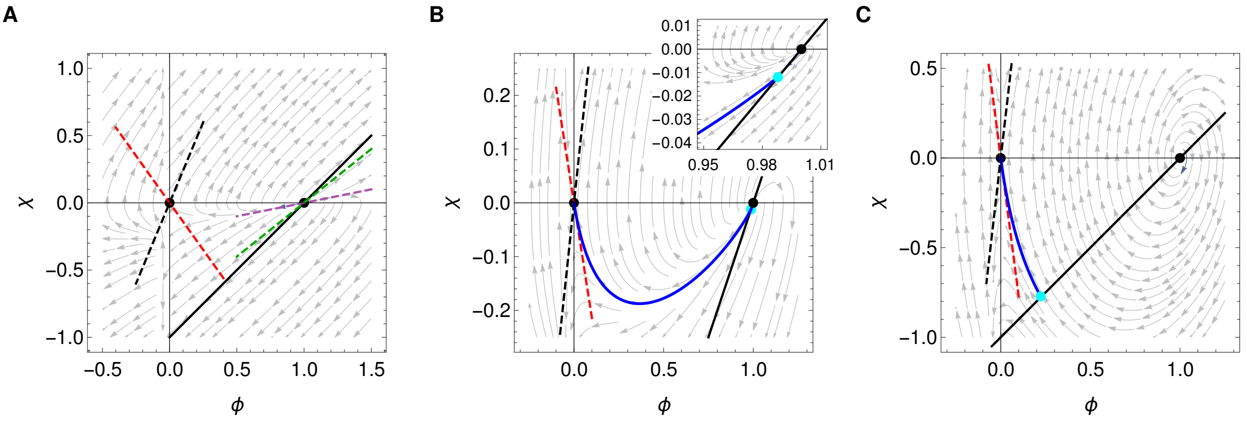

Figure S2: Phase space analysis of the system. **A**: $\lambda = 0.17$ and $\kappa = 0.05$, such that $v > v^*$ (washout). **B**: $\lambda = 0.34$ and $\kappa = 0.05$, such that $v < v^*$ (below washout, but close to it), corresponding to the parameters of Fig. 1B. **C**: $\lambda = 3.36$ and $\kappa = 0.05$, such that $v < v^*$ (substantially below washout). In all panels: Arrows: stationary solutions. Blue curve: stationary solution obtained by numerical integration of PDE Eqs. 1.Black markers: fixed points. Dashed lines: directions of the Jacobian eigenvectors at these fixed points (at $(0,0)$: stable direction in red, unstable one in black; at $(1,0)$ if it is an unstable node (i.e. in **A**): slow unstable direction in purple, fast one in green). Black line: boundary condition in $s = 0$, namely $\chi = \phi - 1$. Cyan dot: Crossing of the numerical solution (blue curve) and the boundary condition (black line). Parameters of the numerical solutions are $D = 0.1, 0.2, 2.0$ cm^2/h in panels **A** , **B**, and **C**, respectively. Other parameters are as in Fig. 1B. The inset in panel **B** shows a close-up in the vicinity of $(1,0)$.

First assume that Eq. S23 is satisfied, ensuring that the Fisher-wave-like solution exists (see Fig. S2A). Then, $(1,0)$ is an unstable node and the smallest eigenvalue of the associated Jacobian is $\ell_- = [1-\sqrt{1-4\lambda/(\kappa+1)}]/2$. This eigenvalue satisfies $0 \leq \ell_- \leq 1/2$, and is associated to the eigenvector $(1, \ell_-)$, yielding a slow direction in the phase space with slope between 0 and 1/2. Close to $(1,0)$, the Fisher-wave-like solution follows this slow direction, before curving upwards to finally approach the stable direction of the saddle point in $(0,0)$. Therefore, it only crosses the line $\chi = \phi - 1$ corresponding to the boundary condition in $s = 0$ (see Eq. S24) at the unstable node $(1,0)$. But this unstable node can only be approached at $s \to -\infty$ in nontrivial solutions, which makes it impossible to find a nontrivial solution satisfying Eq. S24. And indeed, our numerical resolutions confirm that if Eq. S23 is satisfied, bacteria are washed out (see Fig. 1C and section S4).

Let us turn to the opposite case, where $v < v^*$ (see Fig. S2B and C). Then, $(1,0)$ is an unstable spiral, and thus the stationary solution such that $\lim_{s\to-\infty}\phi(s) = 1$ and $\lim_{s\to\infty}\phi(s) = 0$ is oscillatory and comprises unphysical parts where $\phi > 1$ (implying negative numbers of bacteria). However, this spiraling behavior means that this special solution crosses the line $\chi = \phi - 1$ (see Eq. S24) in at least another point, say P, than the unstable node $(1,0)$. Thus, there may be nontrivial and physical solutions satisfying Eq. S24, among the solutions that intersect the line $\chi = \phi - 1$ between $(1,0)$ and P (examples of these intersections are shown in cyan in Figs. S2, S3 and S4). These solutions may look quite different from the Fisher-wave like solution that exists for $v \geq v^*$. In particular, they may possess a value of $\phi(0)$ substantially smaller than 1 and a value of $\phi(\sigma)$ substantially larger than 0. These differences are expected to increase as v is decreased further and further from v^* (i.e. more differences from the Fisher-wave-like solutions are expected in Fig. S2B than in Fig. S2C). Consistently, Fig. 1C demonstrates that profiles with strongest spatial dependence are obtained for v not much below the washout limit $v = v^*$, and Fig. S1 includes several numerical solutions with values substantially different from 1 and 0 at the boundaries $s = 0$ and $s = \sigma$. Fig. S3 shows the phase portraits corresponding to some of these cases, illustrating the diversity of solutions. Nevertheless, in the strongly spatial regime where v is not much below the washout limit $v = v^*$, similarities with the Fisher-wave-like solutions that exist for $v > v^*$ are expected. This case is illustrated e.g. by Fig. S3B.

Here, differences with Fisher waves are brought by the boundary conditions, and in particular by the one at $x = 0$. In addition to the different parameter regime where nontrivial solutions exist, another difference is that all horizontal translations of a traveling wave solution are also solutions in the Fisher wave case [6], while here, they would not satisfy the boundary conditions. The boundary condition at $x = 0$ is quite important as it corresponds to the transition between the small intestine and the colon. Besides, the boundary condition in $x = L$ also affects the solution, in particular by inducing a second washout limit, see Fig. 1C and section S4. However, for sufficiently large $\sigma = Lv/D$, the value of σ has little impact on the solution (see Fig. S14). Fig. S4 shows phase portraits with different values of L, illustrating its impact for small L, and its lack of impact for $L \geq 4$ cm.

Note that traveling waves arising in Fisher-KPP equations Eq. S19 (including those characterizing expanding

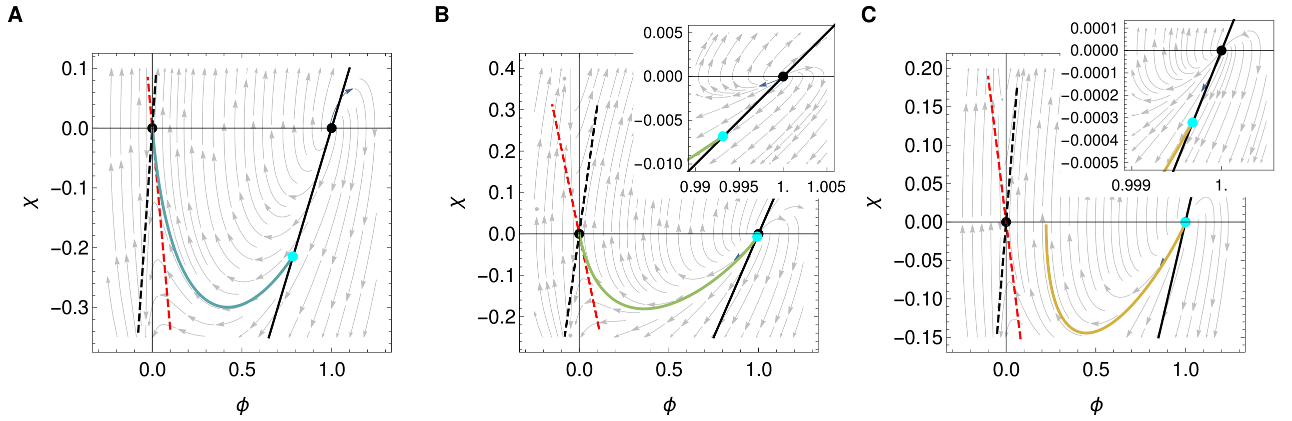

Figure S3: Phase portraits for different types of spatial profiles from Fig. S1B. A: $\lambda = 0.56$ and $\kappa = 0.039$. **B:** $\lambda = 0.32$ and $\kappa = 0.051$. **C:** $\lambda = 0.29$ and $\kappa = 0.054$. Parameters of the numerical solutions are $v = 0.386, 0.509, 0.536$ cm/h in panels **A** , **B**, and **C**, respectively. Other parameters are as in Fig. 1B. Symbols are the same as in Fig. S2 and colors of curves representing numerical solutions match those of Fig. S1B. Insets in panels **B** and **C** are close-ups in the vicinity of $(1, 0)$.

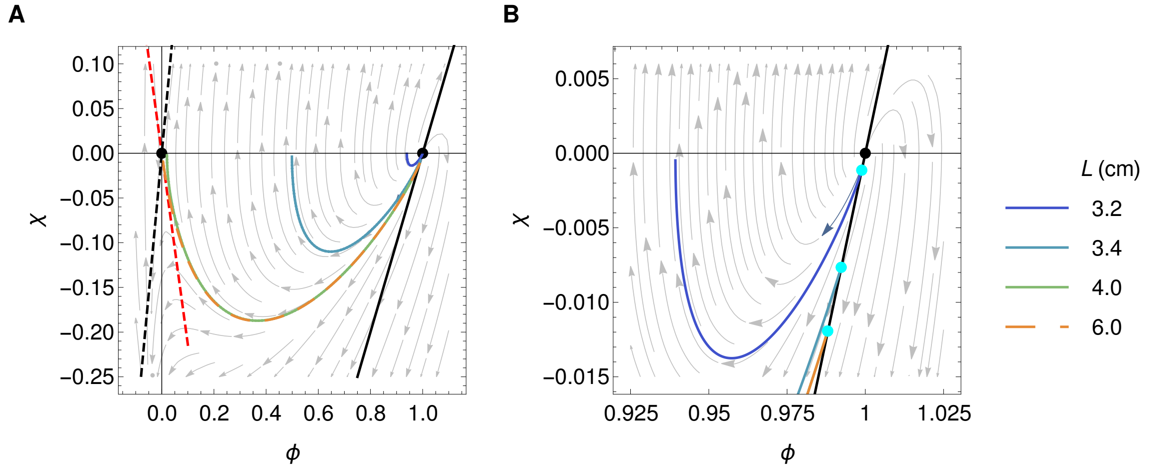

Figure S4: Phase portraits for different system lengths L. All other parameters are as in Fig. 1B. Symbols are the same as in Fig. S2 and colors of curves representing numerical solutions match those of Fig. S11B. Panel **B** is a close-up of panel **A** in the vicinity of $(1, 0)$.

populations) can be either driven by the leading edge (pulled) or by the bulk of the wave (pushed) [8, 13]. Pulled waves have a velocity that depends only on $f(0)$, and are obtained when $f'(u) \leq f'(0)$ for all $u \in [0, 1]$ and $f'(u) = f'(0) + O(h^p)$ for some $p > 0$ when $u \to 0$, in addition to the above conditions $f'(0) > 0$, $f'(1) < 0$, and $f(x) > 0$ for all $x \in]0, 1[$ [16, 17]. These conditions are all satisfied by f in Eq. S21, and thus the associated Fisher-KPP equation would lead to pulled waves. Pushed waves have a velocity that depends on the full nonlinearity of f, and can be obtained when growth rates are nonmonotonic with population density, which can occur with cooperativity (Allee effect) [8, 13]. However, here, the velocity v is an imposed parameter, in contrast to a traveling wave velocity, and the boundary conditions yield further differences, as discussed above.

Note also that it would be interesting to further investigate the stability of solutions to time-dependent perturbations, as was done e.g. for the Fisher wave in the Fisher equation [6]. Here, the analysis would be different as one would need to start from the system of partial differential equations Eqs. 1.

S4 Washout limits

The washout limits are the limits where all bacteria get washed out of the system. Mathematically, they correspond to a bifurcation point in the parameter space where the trivial steady state solution $F(x) = F_{\text{in}}$ (and $B(x) = 0$) becomes stable.

In the chemostat, this limit is easy to determine. There are two steady state solutions, $F^{\text{c}} = kc/(r - c)$ and $F^* = F^{\text{c}}_{\text{in}}$. Eigenvalues of the Jacobian associated to Eqs. S33 and S34 for both steady states change their sign

at same point in the parameter space, namely at

$$c = \frac{r}{k/F_{\text{in}}^{\text{c}} + 1} \equiv c_{\text{wo}}. \tag{S25}$$

The bifurcation scenario is such that at c_{wo} the two steady state solutions collide, change stability, and one (nontrivial) solution disappears, making it a transcritical bifurcation.

In the spatial system, a complete analytical treatment is more difficult, even though one of the washout limits was found by our phase portrait analysis above (see Eq. S23) . Numerically, we find one positive nontrivial solution for $v < v_{\text{wo}}$, where v_{wo} is the bifurcation point which depends on the rest of the system parameters, and only the trivial solution for $v > v_{\text{wo}}$, implying a change of the stability at $v = v_{\text{wo}}$ when the two steady states collide.

It is possible to find analytical estimates for the washout limits in the spatial system by comparing key length scales and time scales , as discussed in Ref. [3]. Let us first consider the case of large diffusion coefficients, when concentration profiles are flat regardless of the value of v. We compare the time τ_{flow} needed for a bacterium to travel through the system from the entrance to the exit to the minimal time τ_{repl} taken by a bacterium to replicate in this system (for $F = F_{in}$). If $\tau_{\text{flow}} < \tau_{\text{repl}}$, i.e.

$$\frac{L}{v} < \frac{\kappa + 1}{r}, \tag{S26}$$

bacteria get washed out, so that an estimate of the washout velocity is

$$v_{\text{wo}} = \frac{rL}{\kappa + 1}, \tag{S27}$$

which is in good agreement with the numerical results, as shown by Figure S5, and Figure 1C in the main text.

Next, let us compare the diffusion and flow characteristic lengths at the time of replication, τ_{repl}. Washout occurs if $L_{\text{diff}} < L_{\text{flow}}$, i.e.

$$\sqrt{2D\tau_{\text{repl}}} < v\tau_{\text{repl}}, \tag{S28}$$

$$\sqrt{2\frac{D(\kappa + 1)}{r}} < \frac{v(\kappa + 1)}{r}, \tag{S29}$$

which gives

$$v_{\text{wo}} = \sqrt{\frac{2rD}{\kappa + 1}}. \tag{S30}$$

This second washout limit is in agreement with Eq. S23, as well as with the numerical results depicted in Figures S5 and 1, up to a factor 2. Indeed, numerically, we find a good agreement with

$$v_{\text{wo}} = v^* = \sqrt{\frac{4rD}{\kappa + 1}}. \tag{S31}$$

The results in Figure S5 are consistent with those of Figure 1C where $(\kappa + 1) \leq 1.25$, and demonstrate the robustness of these results across a wide range of κ values.

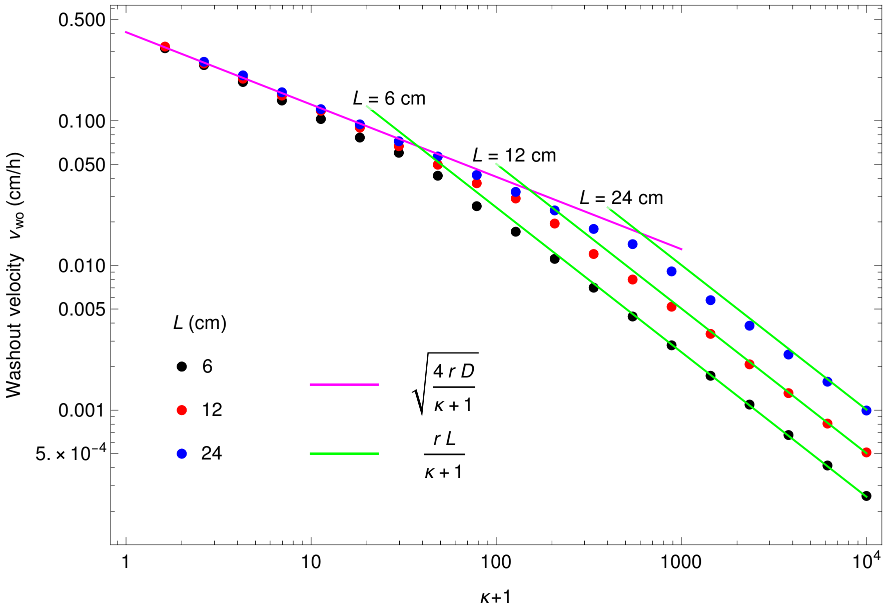

Figure S5: Washout limits in the spatial system as a function of $\kappa + 1$. The two washout limits are obtained by fixing the diffusion coefficient at $0.1\,\mathrm{cm}^2/\mathrm{h}$, and varying the velocity for 20 different values of $\kappa + 1$. The first velocity for which $F(L) > 1. - 10^{-10}$ is recorded as the washout velocity $v_{\rm wo}$. The process is repeated for three different values of length $L = 6, 12, 24$ cm. Other parameters are $v\ F_{\rm in} = 1\,\mathrm{mM\,cm/h}$, $k = \kappa F_{\rm in}$ mM, $r = 0.42\,\mathrm{h}^{-1}$, $\alpha = 6.13 \times 10^8$ bacteria/mM. The green and magenta lines correspond to the two washout limits described by Eqs. S27 and S31, respectively.

S5 Correspondence between the spatial system and the chemostat

S5.1 Main matching condition

We wish to match the total number of divisions occurring in the spatial system and in the chemostat. At stationary state we thus aim to match the amount of food entering and exiting these different systems, as well as the amount of bacteria exiting them.

- Spatial system: amount of food entering: $dN_{\mathrm{F,in}}/dt = F_{\mathrm{in}} vS$; amount of food exiting: $dN_{\mathrm{F,out}}/dt = F(L)vS$; amount of bacteria exiting: $dN_{\mathrm{B,out}}/dt = B(L)vS$;
- Chemostat: amount of food entering: $dN_{\mathrm{F,in}}/dt = F^{\mathrm{c}}_{\mathrm{in}} cV$; amount of food exiting: $dN_{\mathrm{F,out}}/dt = F^{\mathrm{c}} cV$; amount of bacteria exiting: $dN_{\mathrm{B,out}}/dt = B^{\mathrm{c}} cV$.

Here V is the volume of the chemostat, c is the dilution rate i.e. the outflow rate per unit volume of the chemostat. Concentrations in the chemostat are indicated by a superscript c. Meanwhile, v denotes the velocity in the spatial system, S the section and L the length of the spatial system.

Hence, our matching condition reads:

$$\frac{F_{\mathrm{in}}}{F^{\mathrm{c}}_{\mathrm{in}}} = \frac{F(L)}{F^{\mathrm{c}}} = \frac{B(L)}{B^{\mathrm{c}}} = \frac{cV}{vS} \,. \tag{S32}$$

S5.2 Constraints from each separate system

In the spatial system, we have $F(L) = F_{\mathrm{in}}\phi(\kappa, \lambda, \sigma)$ and $B(L) = \alpha\,[F_{\mathrm{in}} - F(L)]$, i.e. $B(L) = \alpha F_{\mathrm{in}}\,[1 - \phi(\kappa, \lambda, \sigma)]$, where ϕ is the dimensionless function introduced above.

In the chemostat, the following equations are satisfied:

$$\frac{dF^{\mathrm{c}}}{dt} = -\frac{r}{\alpha}\frac{F^{\mathrm{c}}B^{\mathrm{c}}}{k + F^{\mathrm{c}}} + cF^{\mathrm{c}}_{\mathrm{in}} - cF^{\mathrm{c}} \,, \tag{S33}$$

$$\frac{dB^{\mathrm{c}}}{dt} = r\frac{F^{\mathrm{c}}B^{\mathrm{c}}}{k + F^{\mathrm{c}}} - cB^{\mathrm{c}} \,. \tag{S34}$$

At stationary state

$$0 = -\frac{r}{\alpha}\frac{F^{\mathrm{c}}B^{\mathrm{c}}}{k + F^{\mathrm{c}}} + cF^{\mathrm{c}}_{\mathrm{in}} - cF^{\mathrm{c}} \,, \tag{S35}$$

$$0 = r\frac{F^{\mathrm{c}}B^{\mathrm{c}}}{k + F^{\mathrm{c}}} - cB^{\mathrm{c}} \,. \tag{S36}$$

If $B^{\mathrm{c}} \neq 0$, this yields $rF^{\mathrm{c}}/(k + F^{\mathrm{c}}) = c$, which means that the dilution rate c of the chemostat sets the effective division rate of the bacteria, a fundamental chemostat property. And then (with $c \neq 0$)

$$F^{\mathrm{c}} = \frac{kc}{r - c} \,, \tag{S37}$$

$$B^{\mathrm{c}} = \alpha\,(F^{\mathrm{c}}_{\mathrm{in}} - F^{\mathrm{c}}) = \alpha F^{\mathrm{c}}_{\mathrm{in}}\left(1 - \frac{k}{F^{\mathrm{c}}_{\mathrm{in}}}\frac{c}{r - c}\right) \,. \tag{S38}$$

Hence, our matching condition Eq. S32 reads:

$$\frac{F_{\mathrm{in}}}{F^{\mathrm{c}}_{\mathrm{in}}} = \frac{F_{\mathrm{in}}\phi(\kappa, \lambda, \sigma)}{kc/(r - c)} = \frac{\alpha F_{\mathrm{in}}\,[1 - \phi(\kappa, \lambda, \sigma)]}{\alpha F^{\mathrm{c}}_{\mathrm{in}}\,[1 - k/F^{\mathrm{c}}_{\mathrm{in}}\, c/(r - c)]} = \frac{cV}{vS} \,, \tag{S39}$$

which reduces to two equations relating the spatial system (left hand-side) to the chemostat (right hand-side):

$$\phi(\kappa, \lambda, \sigma) = \frac{k}{F^{\mathrm{c}}_{\mathrm{in}}}\frac{c}{r - c} \,, \tag{S40}$$

$$vSF_{\mathrm{in}} = cVF^{\mathrm{c}}_{\mathrm{in}} \,. \tag{S41}$$

We assume that the parameters of the spatial system are given. Then we need to choose those of the chemostat in order to have a good matching. The parameters specific to the chemostat are $c, F^{\mathrm{c}}_{\mathrm{in}}, V$. Note that k and r are assumed to be the same in both systems. In principle Eqs. S40 and S41 allow us to fix 2 out of these 3 free chemostat parameters.

S5.3 Additional matching conditions

We may want to impose additional matching conditions between the chemostat and the spatial system:

1. Same total volume: $V = SL$. This implies $\frac{cV}{vS} = \frac{cL}{v}$, and Eq. S32 would be modified accordingly.
2. Same volume exiting per unit time: $cV = vS$. This implies $\frac{cV}{vS} = 1$, and Eq. S32 would be modified accordingly.
3. Same outflow rate relative to the total volume: $c = \frac{vS}{LS} = \frac{v}{L}$. This implies $\frac{cV}{vS} = \frac{V}{LS}$, and Eq. S32 would be modified accordingly.

We note that if we impose two of these three conditions simultaneously, then the third one is also satisfied automatically, but Eqs. S40 and S41 and all three conditions above can be satisfied simultaneously only when $F(L) = vk/(rL - v)$.

S5.4 Properties of the matching chemostats

In general, we can impose only 3 independent conditions, setting the values of $c, F_{\mathrm{in}}^{\mathrm{c}}, V$. Specifically, we have to take Eqs. S40 and S41, plus one of the three conditions numbered 1, 2 and 3 above. These three possibilities are discussed in Table S1 and illustrated in Figure S6.

Matching condition		c	$F_{\mathrm{in}}^{\mathrm{c}}$	V
1. Same total volume	$\frac{cV}{vS} = \frac{cL}{v}$	$\frac{F(L)v}{2Lk}\left(\sqrt{\frac{4Lrk}{F(L)v}+1}-1\right)$	$\frac{F_{\mathrm{in}}v}{2Lr}\left(1+\sqrt{\frac{4Lrk}{F(L)v}+1}\right)$	$L\,S$
2. Same volume exiting per unit time	$\frac{cV}{vS} = 1$	$\frac{F(L)r}{k+F(L)}$	F_{in}	$\frac{v\,S(F(L)+k)}{F(L)r}$
3. Same outflow rate relative to the total volume	$\frac{cV}{vS} = \frac{V}{SL}$	$\frac{v}{L}$	$\frac{F_{\mathrm{in}}kv}{F(L)(Lr-v)}$	$\frac{F(L)\,L\,S(Lr-v)}{kv}$

Table S1: Chemostat parameters $c, F_{\mathrm{in}}^{\mathrm{c}}, V$ as a function of the parameters of the spatial system for three different matching conditions.

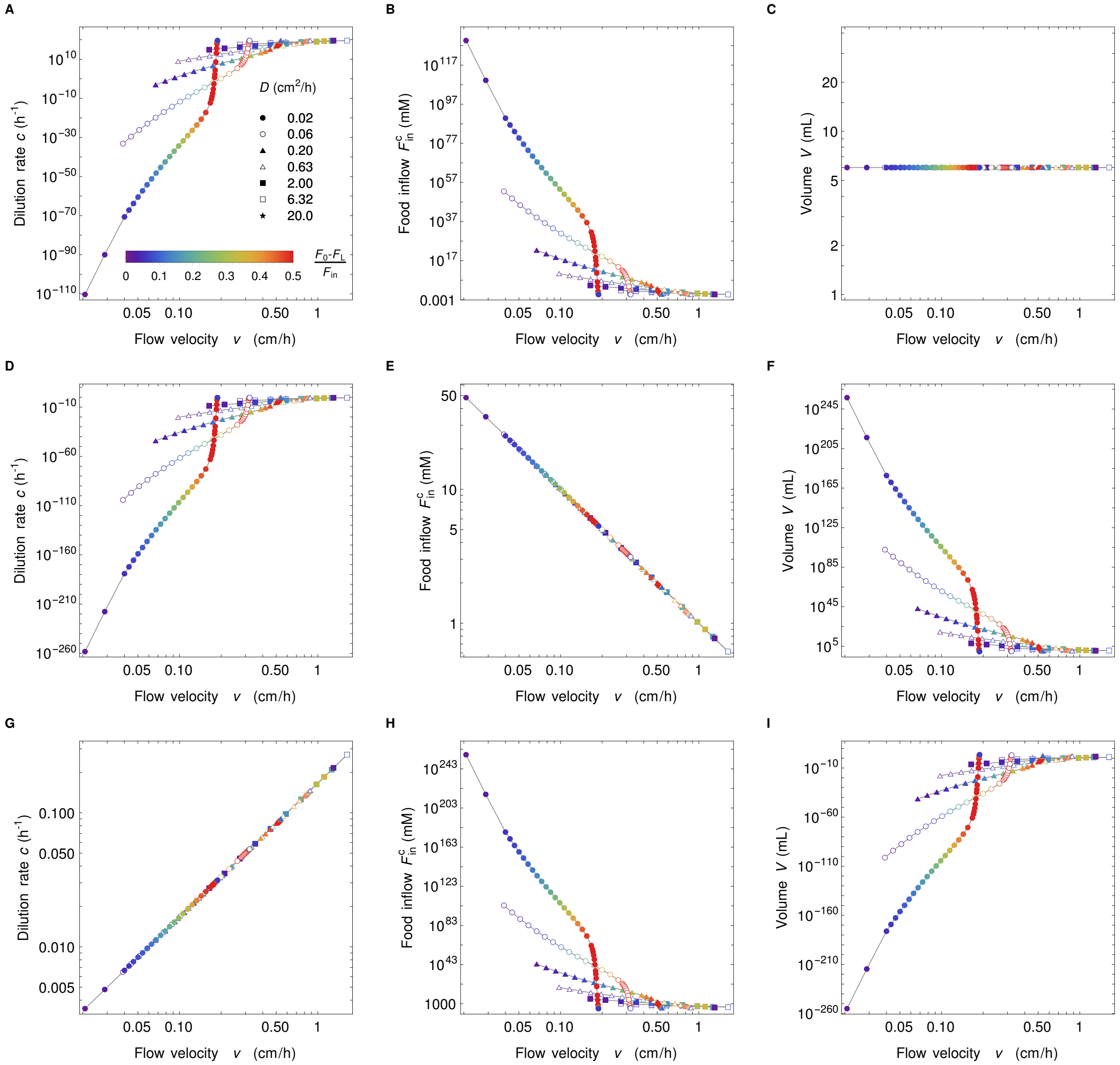

Figure S6: Chemostat parameters. Parameters of the chemostat matching the spatial system in the conditions of Figure 3. Each row in the figure (top to bottom) represents matching condition 1 to 3, while each column in the figure represents a given parameter: dilution rate c (left), food inflow F_{in}^{c} (middle), and volume V (right). Due to the very small values of food concentration exiting the gut, parameters of the chemostat system can have very large or very small values. This is particularly true for smaller diffusion constants and/or small velocities.

Finally, table S2 gives the expression of various useful quantities for the spatial system and for the chemostat.

	Spatial system	Chemostat
Reproduction rate	$\rho(x) = r\dfrac{F(x)}{k+F(x)}$	$\rho^{\mathrm{c}} = r\dfrac{F^{\mathrm{c}}}{k+F^{\mathrm{c}}}$
Reproductions per unit volume and unit time	$R(x) = B(x)\rho(x)$ $= r\alpha\dfrac{F(x)\,[F_{\mathrm{in}}-F(x)]}{k+F(x)}$	$R^{\mathrm{c}} = B^{\mathrm{c}}\rho^{\mathrm{c}}$ $= r\alpha\dfrac{F^{\mathrm{c}}\,(F^{\mathrm{c}}_{\mathrm{in}}-F^{\mathrm{c}})}{k+F^{\mathrm{c}}}$
Total reproduction rate	$N_{\mathrm{R}} = r\alpha\,S\displaystyle\int_0^L \frac{F(x)\,[F_{\mathrm{in}}-F(x)]}{k+F(x)}dx$ $= \alpha v S\,[F_{\mathrm{in}}-F(L)]$	$N^{\mathrm{c}}_{\mathrm{R}} = r\alpha V\dfrac{F^{\mathrm{c}}\,(F^{\mathrm{c}}_{\mathrm{in}}-F^{\mathrm{c}})}{k+F^{\mathrm{c}}}$ $= \alpha c V\,(F^{\mathrm{c}}_{\mathrm{in}}-F^{\mathrm{c}})$
Total population	$N_{\mathrm{T}} = \alpha S\displaystyle\int_0^L [F_{\mathrm{in}}-F(x)]\,dx$	$N^{\mathrm{c}}_{\mathrm{T}} = \alpha V\,(F^{\mathrm{c}}_{\mathrm{in}}-F^*)$
Active population	$N_{\mathrm{A}} = \alpha S\displaystyle\int_0^{x^*} [F_{\mathrm{in}}-F(x)]\,dx, \quad x^* : F(x^*) = k$	$N^{\mathrm{c}}_{\mathrm{A}} = N^{\mathrm{c}}_{\mathrm{T}}$
Fixation probability	$\mathcal{F} = \dfrac{\int_0^L R(x_{\mathrm{M}})\frac{M(x_{\mathrm{M}})}{B(x_{\mathrm{M}})}dx_{\mathrm{M}}}{\int_0^L R(x_{\mathrm{M}})dx_{\mathrm{M}}}$	$\mathcal{F}^{\mathrm{c}} = \dfrac{N_{\mathrm{M}}}{N^{\mathrm{c}}_{\mathrm{T}}}$
Washout limit	$v_{\mathrm{wo}} = \begin{cases} \frac{rL}{k/F_{\mathrm{in}}+1} \\ \sqrt{\frac{4rD}{k/F_{\mathrm{in}}+1}} \end{cases}$	$c = \dfrac{r}{k/F^{\mathrm{c}}_{\mathrm{in}}+1}$

Table S2: Comparison of the main derived quantities in the spatial system and the chemostat.

S6 Early dynamics of mutant bacteria concentration

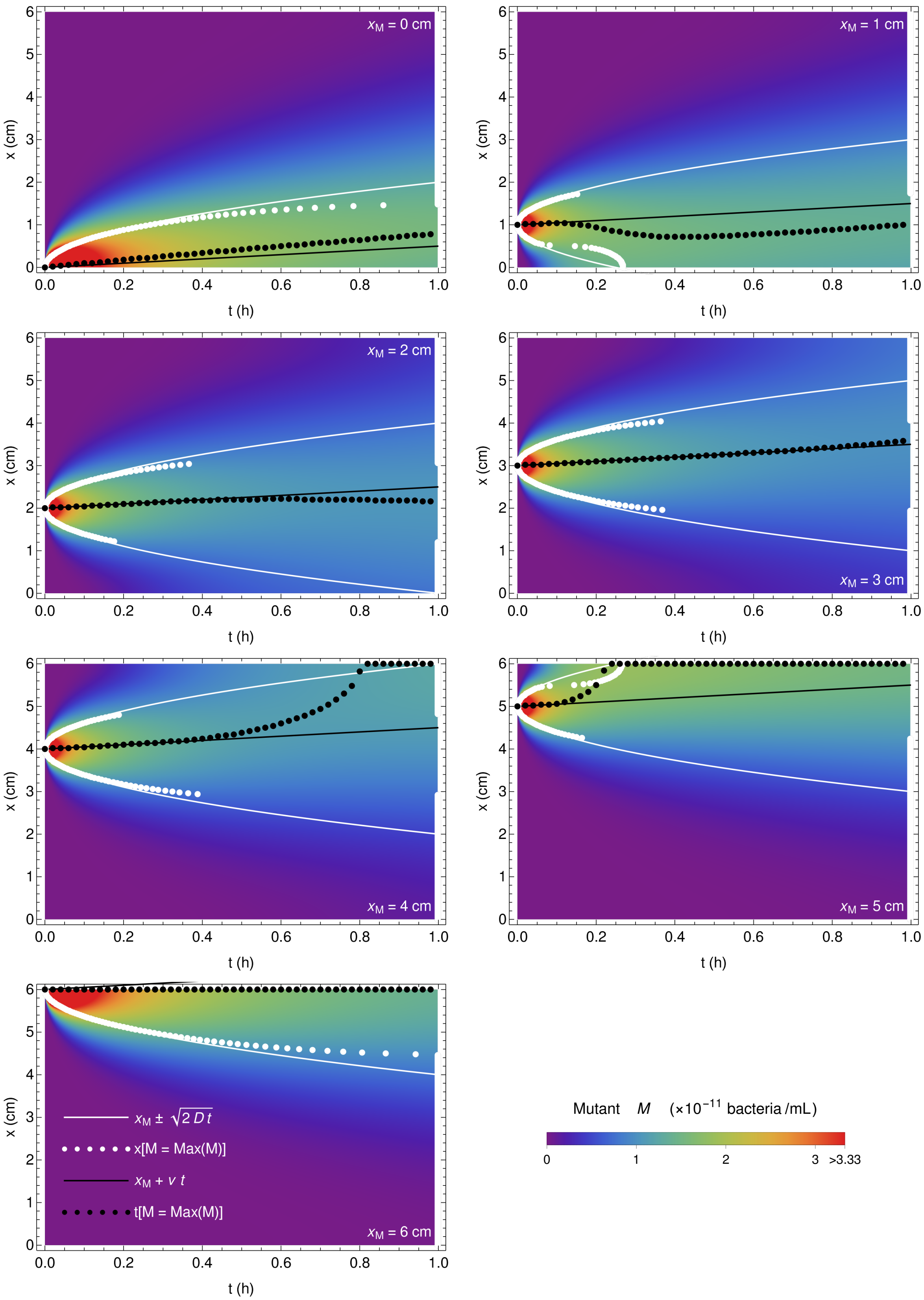

Figure S7: Early dynamics of mutant concentration. Spatio-temporal evolution of mutant concentration for $x_{\mathrm{M}} = 0,\, 1,\, 2,\, 3,\, 4,\, 5,\, 6\,\mathrm{cm}$ in the first hour after mutant introduction. Black points correspond to the maximum mutant concentration at time t, white points correspond to the maximum mutant concentration at position x, black line is $x_{\mathrm{M}} + vt$, and white curve is $x_{\mathrm{M}} \pm \sqrt{2Dt}$. The parameter values are $D = 0.4\,\mathrm{cm}^2/\mathrm{h}$, $v = 0.5\,\mathrm{cm/h}$, $k = 0.1\,\mathrm{mM}$, $r = 0.42\,\mathrm{h}^{-1}$, $v\,F_{\mathrm{in}} = 1\,\mathrm{mM\,cm/h}$, $\alpha = 6.13 \times 10^{8}$ bacteria/mM and $M_0 = 3.33 \times 10^{-9}$bacteria/mL.

S7 Stationary state of mutant bacteria concentration versus the initial position x_M of mutants

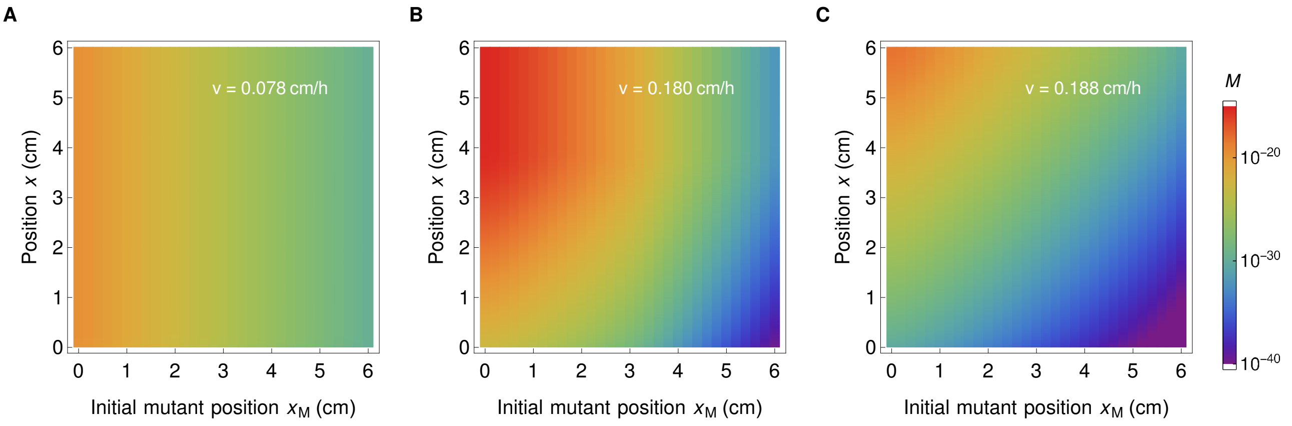

Figure S8: Stationary distribution of mutant bacteria in the gut. Concentration of mutant bacteria as a function of position x, and initial mutant position x_M, for $D = 0.02\,\mathrm{cm}^2/\mathrm{h}$, and for three different values of flow velocity v, corresponding to the flat concentration profile in the well mixed regime (**A**), spatial concentration profile (**B**), and close to the washout limit (**C**). In all three cases, we observe that the final concentration of the mutant bacteria is smaller if the initial position x_M is further along the gut. This figure corresponds to three points of the top curve in Fig. 3.

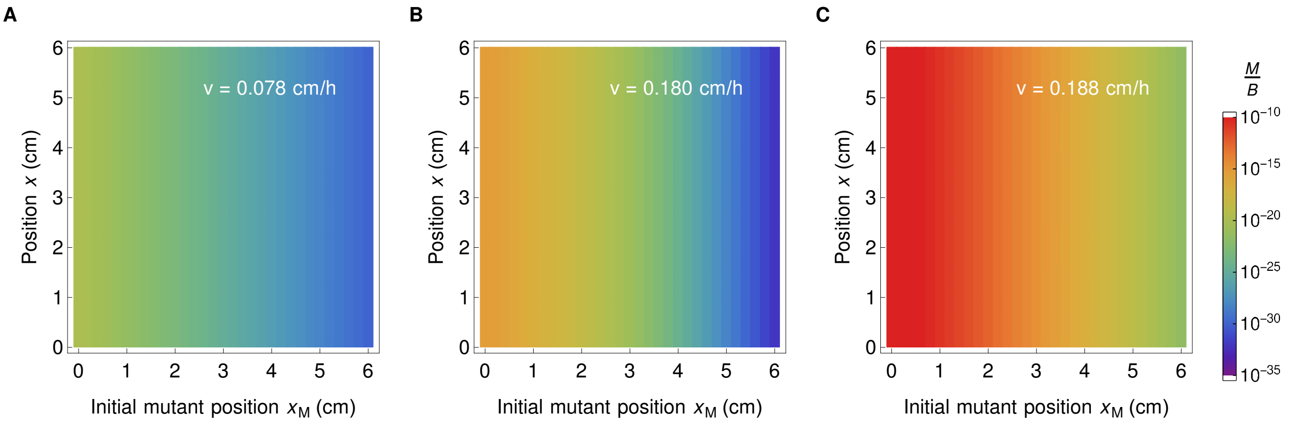

Figure S9: Stationary ratio of mutant to wild type bacteria concentrations. The ratio M/B is shown in all three cases corresponding to Fig. S8. Consistently with our analytical predictions discussed in the main text, the M/B ratio is constant along the gut. In addition, we observe that it monotonically decreases as a function of initial mutant position x_M, and the decrease is the most pronounced in the case of the spatial profile (panel **B**) where the ratio at the beginning and at the end of the gut are several orders of magnitude apart.

S8 Location where most mutants that fix originate

While the position of the maximum of the ratio M/B of mutant to wild type bacteria concentrations is always at the entrance of the gut (see main text and Figure 2), the position of the maximum of the number R of reproduction events per unit volume and unit time depends on parameter values. We find that for flat concentration profiles, it is located either close to the entrance of the gut (for small velocities yielding an almost well-mixed system) or at the exit of the gut (close to the washout limit). Conversely, for spatial concentration profiles, its location is intermediate (see Figure S10A). Because of this, in the regime with strong spatial dependence, we find that the position of the maximum of the product $R\,M/B$ of these two quantities ranges between 0 and $L/2$ (see Figure S10B and C). The position of the latter maximum corresponds to the location where most mutants that fix tend to originate.

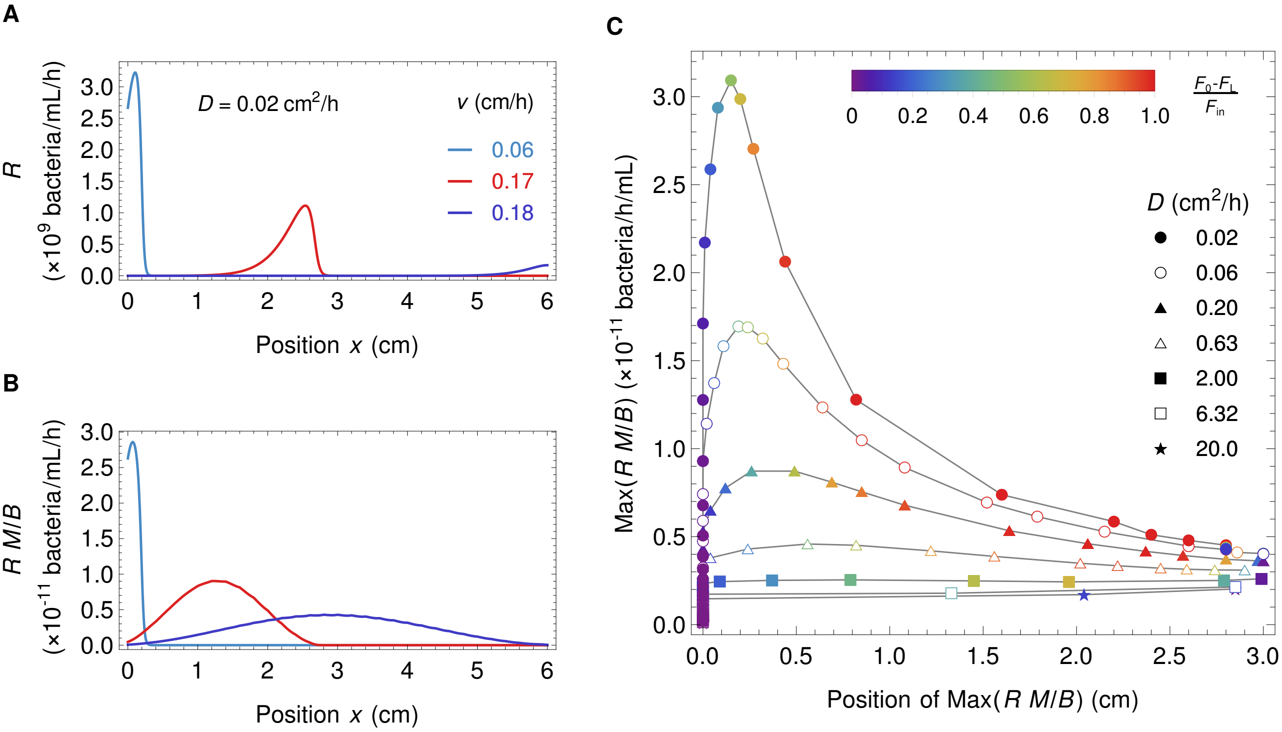

Figure S10: Maximum of R and $R\,M/B$. **A:** Number R of reproduction events per unit volume and unit time as a function of position along the gut for three different types of concentration profiles, almost well-mixed (light blue), spatial (red), and close to the washout limit (purple). **B:** Maximum of the product $R\,M/B$ of the reproduction events per unit volume and unit time and of the ratio of mutant and wild type bacteria as a function of space for the same three concentration profiles as in A (same colors). **C:** Maximum value of $R\,M/B$ as a function of its position for the data set in Figure 3 of the main text.

S9 Calculation of the active population size

The active population corresponds to the bacteria in the zone where reproduction rate is significant. Concretely, it is defined as

$$N_{\mathrm{A}} = S \int_0^{x^*} B(x)dx\,, \tag{S42}$$

where x^* corresponds to the point in the gut segment where the food concentration equals the Monod constant k, i.e. $F(x^*) = k$, implying that the reproduction rate $\rho(x^*)$ is equal to half of the maximal possible reproduction rate, which is obtained if $F(x) \gg k$. Thus,

$$B(x^*) = \alpha[F_{\mathrm{in}} - F(x^*)] = \alpha[F_{\mathrm{in}} - k] = \alpha F_{\mathrm{in}}(1-\kappa)\,. \tag{S43}$$

In the active population thus defined, the reproduction rate of bacteria is at least equal to half of its maximal possible value.

S10 Impact of the dimensionless parameters

S10.1 Holding the dimensionless parameters fixed

To illustrate the relevance of the dimensionless parameters introduced in section S2.2 to describe the stationary profiles, we vary system parameters so that we hold the dimensionless ones fixed. The reference for fixing them is Figure 2:

$$\kappa = \frac{k}{F_{\text{in}}} = \frac{0.1}{2.0} = 0.050, \tag{S44}$$

$$\lambda = \frac{rD}{v^2} = \frac{0.42 \times 0.2}{0.5^2} = 0.34, \tag{S45}$$

$$\sigma = \frac{Lv}{D} = \frac{6 \times 0.5}{0.2} = 15. \tag{S46}$$

We first vary the gut length L in order to ease the discretization of the space. Once the L is chosen, the dimensionless parameters are fixed by adjusting v, and then F_{in}, to keep the product $v\,F_{\text{in}}$ constant. Other parameters are kept fixed. Figure S11A shows the corresponding concentration profiles. They all have the same shape, as evidenced by rescaling the food concentration by F_{in} and the spatial coordinate by L (see inset of the Figure S11A). Figure S11B shows that the fixation probability for these profiles scales with active population, $\mathcal{F} = N_{\text{M}}/N_{\text{A}}$, consistently with our expectations, since the concentration profiles are strongly spatially dependent.

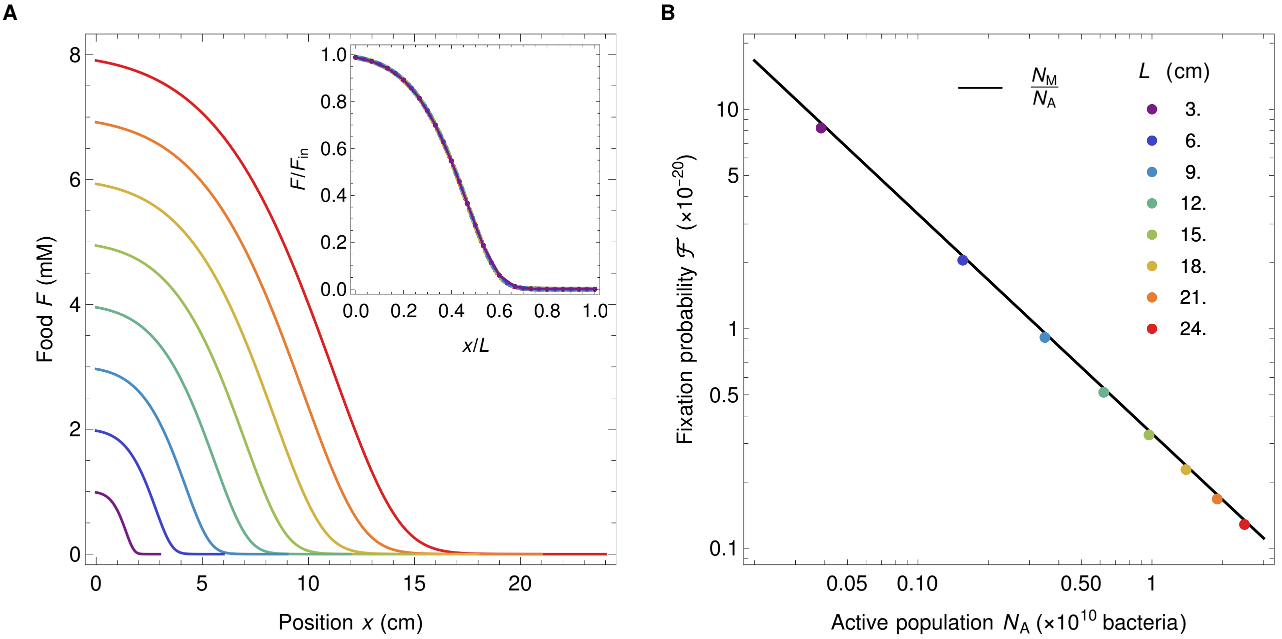

Figure S11: **A** Food concentration profiles for different system sizes and keeping nondimensional parameters, λ, κ, σ, fixed. The profiles can be rescaled by dividing food concentration by F_{in} and space by L (see inset of A) showing all the profiles are identical up to the scaling factors. **B** Fixation probability for the profiles in panel **A**. The parameters are $D = 0.2\,\text{cm}^2/\text{h}$, $v = 3./L$ cm/h, $F_{\text{in}} = 1./v$ mM, $k = 0.05/v$ mM, $r = 1.68 \cdot v^2$ h^{-1}, $\alpha = 6.13 \times 10^8$ bacteria/mM, $N_{\text{M}} = 3.33 \times 10^{-11}$ bacteria and the L values are listed in the panel **B**.

S10.2 Varying each dimensionless parameter

Finally, we systematically investigate the impact of each dimensionless parameter by varying one of them while holding the other two fixed. We keep parameters v, D and F_{in} fixed throughout, and vary k, r, and L one at a time in order to change κ, λ, and σ, respectively. Results in Figures S13, S12 and S14 show that the fixation probability is well described by $\mathcal{F} = N_{\text{M}}/N_{\text{A}}$ except in cases where the food concentration profile is less spatial because food is substantially depleted even at the entrance of the gut, which occurs for the four largest values of r in Figure S13.

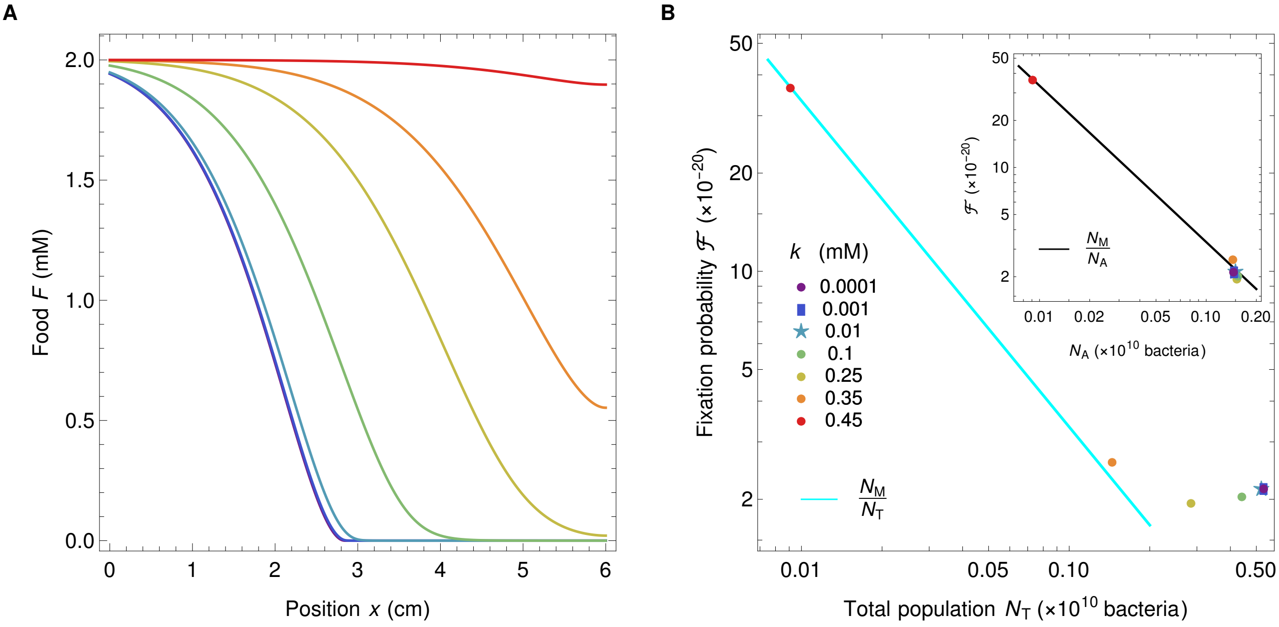

Figure S12: Varying $\boldsymbol{\kappa}$. **A** Food profiles for different values of κ and fixed $\lambda = 0.336$ and $\sigma = 15$. **B** Fixation probability as a function of total population N_{T} and active population N_{A} (in the inset). Different colors correspond to different values of κ (k) and different symbols are used for overlapping data points. Parameters are $v = 0.5\,\text{cm/h}$, $D = 0.2\,\text{cm}^2/\text{h}$, $L = 6.0\,\text{cm}$, $r = 0.42\,\text{h}^{-1}$, $\alpha = 6.13 \times 10^8$ bacteria/mM, and $N_{\text{M}} = 3.33 \times 10^{-11}$ bacteria. k values are listed in the panel **B**.

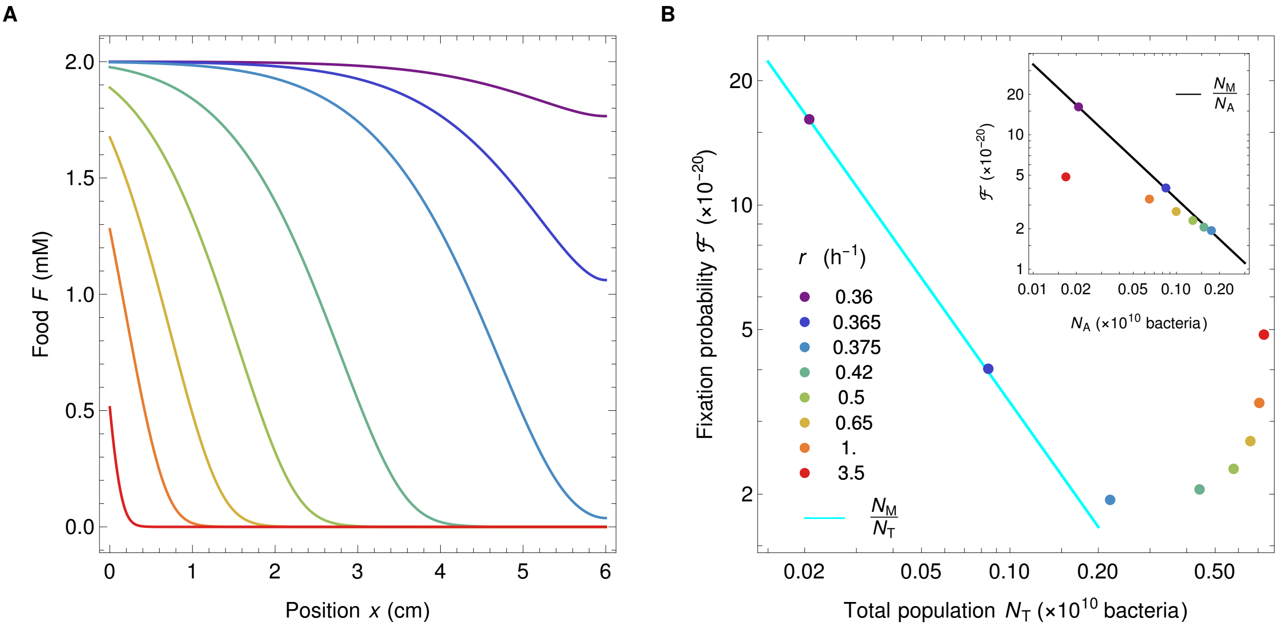

Figure S13: Varying $\boldsymbol{\lambda}$. **A** Food profiles for different values of λ and fixed $\kappa = 0.05$ and $\sigma = 15$. **B** Fixation probability as a function of total population N_{T} and active population N_{A} (in the inset). Different colors correspond to different vales of λ (r). Parameters are $v = 0.5\,\text{cm/h}$, $D = 0.2\,\text{cm}^2/\text{h}$, $k = 0.1\,\text{mM}$, $k = 0.1\,\text{mM}$, $\alpha = 6.13 \times 10^8$ bacteria/mM, and $N_{\text{M}} = 3.33 \times 10^{-11}$ bacteria. r values are listed in the panel **B**.

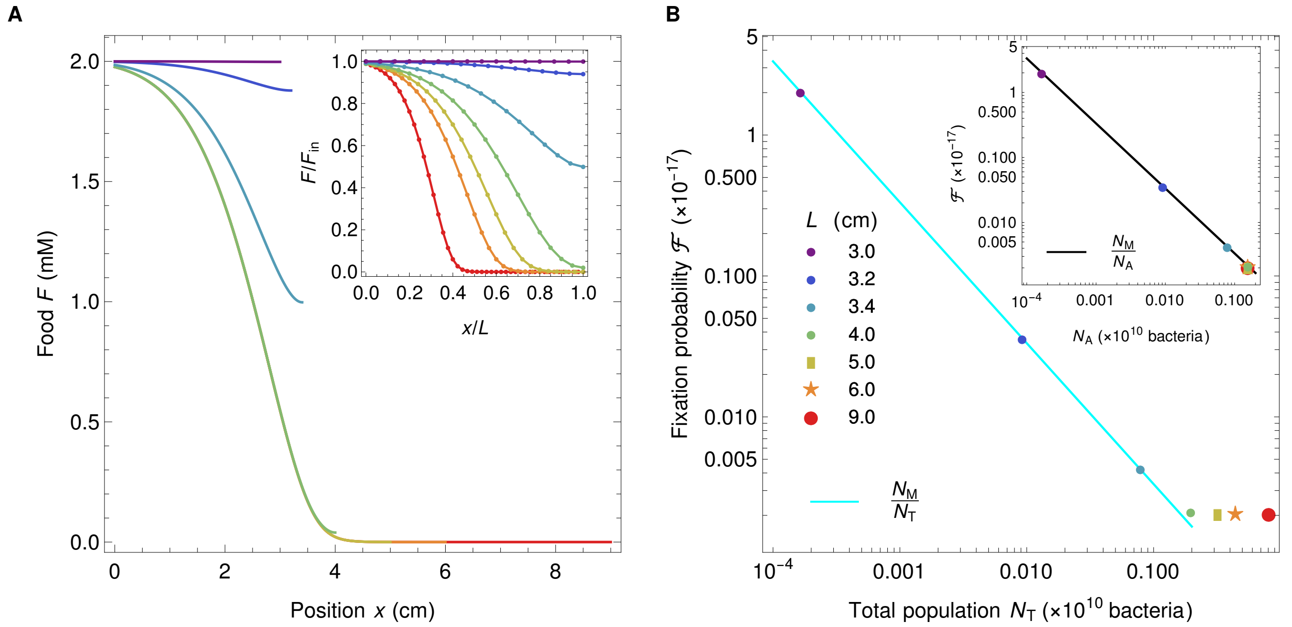

Figure S14: Varying σ. **A** Food profiles for different values of σ and fixed $\lambda = 0.336$ and $\kappa = 0.05$. The inset shows rescaled food profiles, for easier comparison of the spatial dependence. **B** Fixation probability as a function of total population N_{T} and active population N_{A} (in the inset). Different colors correspond to different values of σ (L) and different symbols are used for overlapping data points. Parameters are $v = 0.5\,\mathrm{cm/h}$, $D = 0.2\,\mathrm{cm^2/h}$, $L = 6.0\,\mathrm{cm}$, $r = 0.42\,\mathrm{h^{-1}}$, $\alpha = 6.13 \times 10^8$ bacteria/mM, and $N_{\mathrm{M}} = 3.33 \times 10^{-11}$ bacteria. k values are listed in the panel **B**.

S10.3 Range of the dimensionless parameters and relevance for the human colon

In this section, we discuss the range of values of the dimensionless parameters κ, λ and σ, which are the only parameters of the model that may change the behavior of the system and affect our conclusions (recall that α is just a scaling factor that does not affect spatial profiles and conclusions, see section S1.3). We first summarize the range of values that these dimensionless parameters take in our study and in Refs. [3, 18]. Next, we discuss the realistic range of values that they can take in the human colon, and show that the range studied here is relevant for the human colon .

The parameter values employed in this paper correspond to the mini gut described in [3], where it has been proven that the mathematical model describes well the experimental setup. In [18], the same model was used to describe microbiota growth and composition in the human colon, and parameter values were thus altered to match the properties of the human gut, which is several times bigger than the mini gut. However, this change of parameter values did not significantly modify the values of the dimensionless parameters. As illustrated in Figure S11, holding the dimensionless parameters fixed fully preserves the properties of the system, including the spatial profiles of concentrations, and our conclusions on the active population remain true. Table S3 summarizes the range of dimensionless parameter values considered in Figure 3 of this paper, and compares them to those of Refs. [3, 18].

Dimensionless parameter	Figure 3	Figure 3; $\frac{F(0)-F(L)}{F_{\mathrm{in}}} > 0.9$	Ref. [3]	Ref. [18]
$\kappa = \dfrac{k}{F_{\mathrm{in}}}$	$10^{-4} - 0.21$	$0.014 - 0.083$	0.05	2.5×10^{-4}
$\lambda = \dfrac{rD}{v^2}$	$0.19 - 8.4 \times 10^6$	$0.24 - 0.42$	$0.036 - 9.3$	0.29
$\sigma = \dfrac{Lv}{D}$	$3 \times 10^{-4} - 57$	$7.59 - 56$	$1.5 - 25$	9.6

Table S3: Dimensionless parameter values used in Figure 3 of this paper and in Refs. [3] and [18].

Let us now discuss the realistic range of values of the dimensionless parameters κ, λ and σ in the human colon. Let us start by considering $\kappa = k/F_{\mathrm{in}}$, and for this, let us first estimate F_{in}. Bacteria in the large intestine consume a mix of different nutrients that have not (or not completely) been absorbed in the small intestine. A

large component of them are fibers, and a human typically ingests 25 to 100 g a day of fibers [19]. The colon input also includes a smaller or similar quantity of unabsorbed sugars and starch [20]. Given that the inflow of digesta in the colon is about 2 liters per day [21], the order of magnitude of the incoming food concentration $F_{\rm in}$ in the colon is in the range of 15 to 100 g/L. The Monod constant k depends on many factors, including the substrate and the bacterial strain. Even for glucose, it can range from 0.03 to 5 mg/L depending on how well adapted the bacteria are to growing on glucose [22], and it is typically higher for other substrates (20 to 300 mg/L for acetate [23]; 5 to 900 mg/L for different substrates [24]). Realistic values for $\kappa = k/F_{\rm in}$ are thus in a wide range, but in all cases, they are much smaller than 1, at least as small as 0.1. In the next section where dimensionless parameter values are systematically varied, Figure S12 demonstrates that such values of κ give very similar outcomes, and all results collapse in the limit $\kappa \to 0$.

The two other dimensionless parameters, λ and σ, both involve the effective diffusion coefficient D, which models mixing and is thus hard to measure directly. However, it is empirically well demonstrated that there is a strong gradient of bacterial concentration along the colon [25, 26], and that most nutrients that could be used by bacteria are consumed by the end of the gut [20]. This requires that $\lambda = rD/v^2$ be larger than 1/4 (washout limit, see section S4), and less than a few units (since above, diffusion is strong enough for the system to be almost well mixed). This can be seen on Figure 1C. Accordingly, Table S3 demonstrates that despite the wide range of parameters used in our study, the range of λ is very narrow in the regime that yields strongly spatial profiles (specifically, λ is between 0.24 and 0.42 when $[F(0) - F(L)]/F_{\rm in} > 0.9$). This is in line with the estimate from [18].

Let us finally turn to σ. We notice that $\sigma = Lv/D = Lr/(v\lambda)$. Avoiding washout requires $L/v > (\kappa+1)/r \gtrsim 1/r$ (see section S4), where the last inequality is rather tight because $\kappa \ll 1$. Since in addition $1/\lambda$ is of the order of 2 to 4 in the very spatial regime, $\sigma = Lr/(v\lambda)$ then has to be greater than a few units. Another way to estimate σ is to employ $Lr/v = \tau_{\rm dig}/\tau_{\rm rep}$ where $\tau_{\rm rep} = 1/r$ is the minimal bacterial replication time (within the colon), and $\tau_{\rm dig} = L/v$ is the typical time spent in the system. While the total transit time ranges from one to several days [27], the time spent by the digesta in the ascending colon, which is the upstream part of the colon where the strong gradients of food and bacterial concentrations are located [18], is substantially shorter, of the order of 4 hours [28], and thus $\tau_{\rm dig} \approx 4$ h. Let us now estimate $\tau_{\rm rep}$. Feces weigh about 130 g/day and contain 25–50% of bacteria in mass [19]. The typical mass of a bacteria being 1 pg, this means that about 3–6$\times 10^{13}$ bacteria per day are lost in feces, to be compared with about 4×10^{13} bacteria in the colon [29], leading to about one renewal per day, which means that $\tau_{\rm rep} \leq 24$ h. However, as it is likely that bacteria actively replicate only in the upper part of the colon, while r represents the maximal reproduction rate in the gut, the actual value of $\tau_{\rm rep}$ is expected to be substantially smaller than this upper bond. A lower bound for $\tau_{\rm rep}$ is given by the minimal doubling time of fast replicating bacteria such as *Escherichia coli* in good conditions, which can be as low as 20 minutes [30]. To summarize, $\sigma = \tau_{\rm dig}/(\lambda\tau_{\rm rep})$, with $1/\lambda$ of the order of 2–4 and $\tau_{\rm dig}/\tau_{\rm rep} \gtrsim 1$, and $\tau_{\rm dig}/\tau_{\rm rep} \approx 10$ when considering the ascending colon and the maximal replication rate. This matches well the range of values of σ considered in the present work (see Table S3).

S11 Relevance of neutral mutations

In this study, we focused on neutral mutations, as a first step towards understanding evolution in the gut. What fraction of spontaneous mutations is expected to be effectively neutral in gut bacteria? Despite the importance of beneficial mutations for adaptation, they are expected to be a small fraction of spontaneous mutations, at least in reasonably well-adapted organisms. Therefore, let us introduce the fitness cost δ of a mutation, defined by $f_M/f_W = 1 - \delta$ where f_M is mutant fitness and f_W wild-type fitness (beneficial mutations then have a negative δ). Mutations are effectively neutral if their fitness cost satisfies $N|\delta| \ll 1$ where N is the effective population size [31], which is the active population size here.

Let us estimate active population size in the human colon, where the total bacterial population is of the order of 4×10^{13} bacteria [29]. The total rate of bacterial divisions in the gut is given by the ratio of the total population size to the renewal time. This rate can also be expressed as the active population size divided by the minimal replication time $\tau_{\rm rep}$, because bacteria in the active population reproduce at rates close to the maximal one. Thus, the ratio of active to total population size is equal to the ratio of minimal replication time to renewal time, which, as discussed in previous section, can be as small as $20/(24 \times 60) = 1.4 \times 10^{-2}$, and is likely to be of order 0.1 (also consistent with [32]). In addition, fixation of a mutant occurs within a strain. Different strains may compete for resources, but here, for simplicity, we do not consider this aspect, which is possible if diversity is stable or if different strains employ different resources. There are at least tens of thousands of bacterial phylotypes in the gut, exact diversity characterizations being limited by sequencing depth [33]. Among all these types, some are much more abundant than others, and multiple rare types exist. Let us consider an active population size of order $N = 10^7$ bacteria.

Let us now discuss the range of δ values. Estimating the full distribution of fitness effects of mutations is difficult [34], and many studies focus on beneficial mutations [35] because of their importance in adaptation. A recent microfluidic mutation accumulation experiment [36] performed in *Escherichia coli* allowed to suppress the effect of selection and to study all spontaneous mutations. The average fitness cost $\langle\delta\rangle$ was found to be 3×10^{-3}, but it largely arises from a minority of strongly deleterious mutations. The skewness of the distribution of fitness costs was found to be positive and large, of about 17. Accurate measurements of extremely small fitness effects are highly challenging, but a Gamma distribution of the same mean and skewness would yield 10% of all mutations with a fitness cost δ smaller than 10^{-7}. Thus, a substantial fraction of all spontaneous mutations occurring in gut bacteria is expected to be effectively neutral.

S12 Validation by stochastic simulations

S12.1 Stochastic simulation methods

In our stochastic simulations, space and time are discretized. We denote the discrete steps in space and time used in these stochastic simulations by δx and δt. Recall that our model is one-dimensional and therefore the only spatial dimension to be considered is x, along the gut main axis. To simplify notations, in this section we denote by $F(x)$ the linear concentration of food and by $B(x)$ that of bacteria (which amounts to taking a gut section with unit area).

Discretizing transport. To represent transport in our discrete simulations, rates of exchange of food and bacteria between adjacent segments of lengths δx are defined.

First, to represent diffusive flow, let us introduce the rate r_d at which food moves from one segment to each one of its two closest neighboring segments (upstream or downstream). For the discrete model to converge to our continuous equations as $\delta x \to 0$, r_d has to satisfy:

$$\lim_{\delta x\to 0} r_d\,\delta x\,[F(x+\delta x)-F(x)] = D\frac{\partial F}{\partial x}\,, \tag{S47}$$

where we expressed the diffusive flow per unit time from the segment at x to its immediate downstream neighbor both in the discrete and in the continuous descriptions. This leads to:

$$r_d = \frac{D}{\delta x^2}\,. \tag{S48}$$

Similarly, to represent convective flow, let us define the rate r_v at which food moves from one segment to its downstream neighbor. For the discrete model to converge to our continuous equations as $\delta x \to 0$, r_v has to satisfy:

$$\lim_{\delta x\to 0} r_v\,\delta x\,F(x) = v\,F(x)\,, \tag{S49}$$

where we expressed the convective flow per unit time from the segment at x to its immediate downstream neighbor both in the discrete and in the continuous descriptions. This leads to:

$$r_v = \frac{v}{\delta x}\,. \tag{S50}$$

Then overall, in the discrete model, during δt, the amount of food moving from one segment to its upstream neighbor is $r_-\delta t F(x)\delta x$ where r_- is the rate of upstream transport:

$$r_- = r_d = \frac{D}{\delta x^2}\,. \tag{S51}$$

Meanwhile, the amount of food moving from one segment to its downstream neighbor is $r_+\delta t F(x)\delta x$ where r_+ is the rate of upstream transport:

$$r_+ = r_d + r_v = \frac{D}{\delta x^2} + \frac{v}{\delta x}\,. \tag{S52}$$

Note that the mapping with the continuous equations is obtained for $\delta x \to 0$, and thus results may deviate for finite δx.

Similarly, during δt, individual bacteria have a probability $r_-\delta t$ to jump upstream, and $r_+\delta t$ to jump downstream.

Discretizing growth. Let us start from our continuous equations on concentrations of bacteria and food, and map them to a description with discrete bacteria. Recall that in this section, B and F are linear concentrations of bacteria and food. Restricting to the terms of Eq. 1 modeling growth, we have:

$$\begin{cases} \dfrac{dB}{dt} &= rB\dfrac{F}{F+k}\,, \\ \dfrac{dF}{dt} &= -\dfrac{1}{\alpha}rB\dfrac{F}{F+k}\,. \end{cases} \tag{S53}$$

Denoting by $b(x) = B(x)\delta x$ (resp. $f(x) = F(x)\delta x$) the average number of bacteria (resp. the quantity of food in moles) present in the segment $[x, x+\delta x]$, these equations become:

$$\begin{cases} \dfrac{db}{dt} &= rb\dfrac{f}{f+\tilde{k}}\,, \\ \dfrac{df}{dt} &= -\dfrac{1}{\alpha}rb\dfrac{f}{f+\tilde{k}}\,, \end{cases} \tag{S54}$$

$\tilde{k} = k\delta x$. Similarly, let us introduce $f_{\text{in}} = F_{\text{in}}\delta x$.

Each individual bacterium has a reproduction rate of $rf(x)/[f(x)+\tilde{k}]$, so during δt it has a probability $r\delta t\, f(x)/[f(x)+\tilde{k}]$ to reproduce. If one bacterium reproduces, then $f(x)$ is decreased by $1/\alpha$, thereby ensuring the mapping with Eqs. S54. Note that, since this quantity is fixed, for small values of δx, it may lead to transient negative local values of $f(x)$, due to discretization and to the assumption that food is only consumed in the segment where replication occurs. When this happens, we set the local reproduction rate of bacteria to zero, and thanks to diffusion, such unrealistic states are short-lived.

Simulation steps.

- Initialization: The initial discrete numbers $n_b(x)$ of bacteria in each segment $[x, x+\delta x]$ are obtained from the steady-state numerical solution $B(x)$ of the continuous equations through $n_b(x) = \lceil b(x) \rceil = \lceil B(x)\delta x \rceil$, where $\lceil . \rceil$ denotes the ceiling function.
- Equilibration without mutants:
  1. During δt, a bacterium in $[x, x+\delta x]$ has a probability $rf(x)/[f(x)+\tilde{k}]$ of reproducing. For each bacterium, a random number is drawn in a uniform distribution on the interval $[0, 1]$, and compared to the reproduction probability. This determines whether this bacterium reproduces.
  2. For each bacterium within $[x, x+\delta x]$ that reproduces, the quantity of food $f(x)$ is decreased by $1/\alpha$.
  3. After the steps above, food is updated according to the following rule: $f(x) \leftarrow f(x)\,[1-(r_+ + r_-)\delta t] + f(x+\delta x)r_-\delta t + f(x-\delta x)r_+\delta t$, where the rates r_- and r_+ are given respectively by Eq. S51 and Eq. S52. On the boundaries, $f(0) \leftarrow f(0)\,[1-(r_+ + r_-)\delta t] + f(\delta x)r_-\delta t + [vf_{in}/\delta x + r_- f(0)]\,\delta t$, and $f(L) \leftarrow f(L)\,(1-r_+\delta t) + f(L-\delta x)r_+\delta t$.
  4. For each bacterium at each site x a random number is drawn from a uniform distribution on an interval $[0, 1]$, and compared to the probability to move to the right, $r_+\delta t$, to move to the left, $r_-\delta t$, or to stay, $1-(r_+ + r_-)\,\delta t$. Bacteria move accordingly.

  The above steps 1 to 4 are repeated until time t reaches 1000 hours. A new initial state of bacteria and food is obtained by taking the average in the time interval $t \in [100, 1000]$ h. Then, steps 1 to 4 are repeated for 100 more hours.
- Mutation: At the next replication of one bacterium, we assign to it one mutant daughter bacterium. If more than one bacterium replicates during δt, the mutant daughter bacterium is assigned to one of them, chosen uniformly at random.
- Evolution to fixation or extinction: The above steps 1 to 4 are repeated until extinction or fixation of the mutant bacteria, keeping track of what bacteria are mutant and wild-type. 141,681 different stochastic replicates were run.

Choice of the discretization parameters. Three parameters describe the discrete nature of this stochastic simulation: the discrete spatial step δx, the discrete time step δt, and the finite total number of bacteria $N_{\text{T}} = \sum_x b(x) = \int_0^L B(x)dx$. In the numerical resolutions of the main text, we focused on large bacterial population sizes. But here, as we aim to validate the results from our deterministic model using a stochastic one, we consider less realistic small population sizes, which allows for shorter computation times. However, N_{T}

should not be too small in order to avoid spontaneous extinctions of bacteria due to fluctuations within the time of the simulations. In practice, to control population size, we tune the conversion factor α between food and bacteria. In addition, taking δx or δt not small enough leads to shifts in the food profile compared to the continuous case, as demonstrated in Fig. S15.

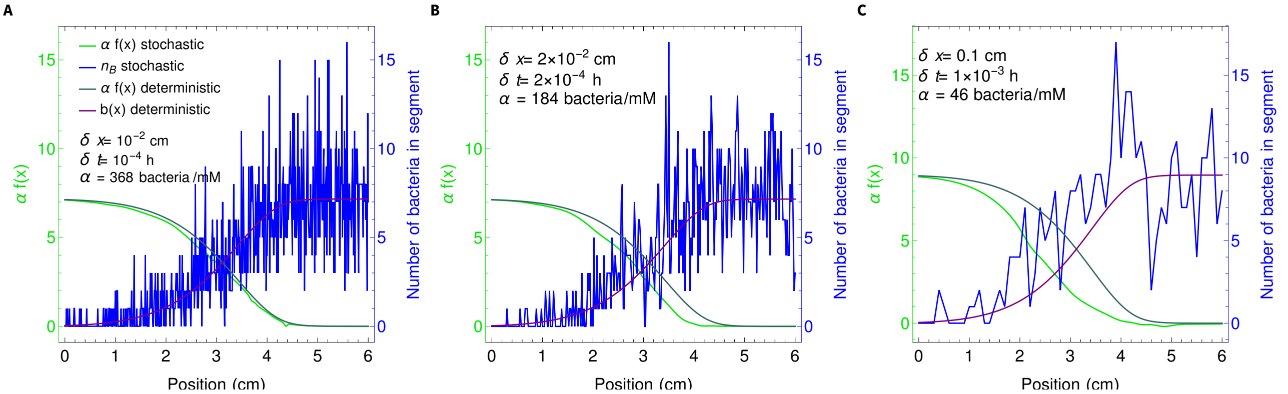

Figure S15: Dependence of the stochastic profiles on the discretization parameters, $\boldsymbol{\delta x}$, $\boldsymbol{\delta t}$, and $\boldsymbol{N_{\mathrm{T}}}$ (via $\boldsymbol{\alpha}$). Numbers of bacteria and rescaled food quantities at time 10^3 h are shown in the stochastic and in the deterministic description versus position x in the gut. Initial conditions are the same in all panels and are taken as described in the "Initialization" item of our simulation steps. The only difference between panels is the value of the discretization parameters. Other parameters of the system are as in Fig. 1B, apart from $v = 0.514$ cm/h (and α which is specified in each panel).

In order to keep simulation times reasonably short, we chose the parameters corresponding to Fig. S15B. Note that this case exhibits a small shift in the spatial profile compared to the continuous case. However, this shift is stable, the steady state relation $B = \alpha(F_{in} - F)$ holds on average, and the source of the shift with respect to the continuous case is known to be the choice of δx and δt. The fluctuations caused by the choice of N_{T} (via α) in Fig. S15B are also stable and are not causing extinctions or changes of spatial regimes.

S12.2 Stochastic simulation results

Here, we present results obtained over 141,681 different stochastic replicates of the simulation described above, with discretization parameters corresponding to Fig. S15B.

Fig. S16 shows the fate of neutral mutants appearing at various locations in the gut. We observe a good agreement between panels A and B, obtained from our stochastic simulations, and panels C and D, obtained from our numerical resolution of the deterministic system (analogous to Fig. 2). In particular, the black curve in Fig. S16A, showing the proportion $p(x_{\mathrm{M}})$ of mutant bacteria that fix versus their introduction position x_{M}, matches the black curve in Fig. S16C, which shows the steady-state ratio M/B of mutant to wild-type bacteria concentrations. This corroborates the fact that the deterministic steady-state ratio M/B in the deterministic description behaves as the mutant fixation probability in the stochastic case, for each given mutant introduction position x_{M}.

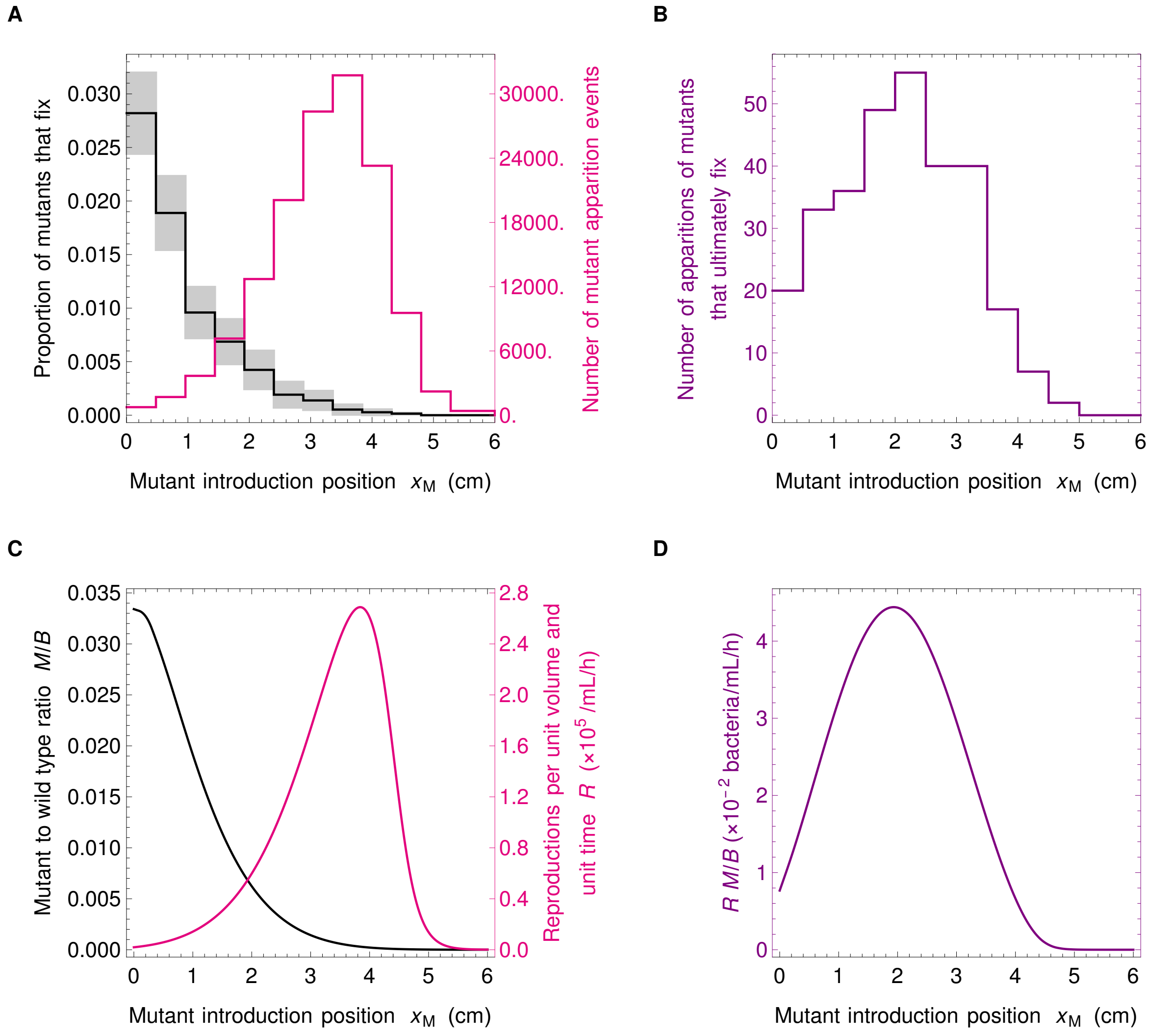

Figure S16: Fate of neutral mutants appearing at various locations in the gut: stochastic and deterministic descriptions. A: Black: proportion $p(x_{\mathrm{M}})$ of mutant bacteria that fix versus their introduction position x_{M}, computed as $p(x_{\mathrm{M}}) = N_{MF}(x_{\mathrm{M}})/N_{MA}(x_{\mathrm{M}})$, where $N_{MF}(x_{\mathrm{M}})$ is the number of mutants that fix in the segment at x_{M}, while $N_{MA}(x_{\mathrm{M}})$ is the number of mutants that appeared in that bin. Gray shaded boxes correspond to $p(x_{\mathrm{M}}) \pm \sqrt{N_{MF}(x_{\mathrm{M}})}/N_{MA}(x_{\mathrm{M}})$. Pink: histogram of the number of mutant apparition events at each introduction position x_{M}. **B:** Histogram of the number of apparition events of mutant bacteria that eventually fix versus their introduction position x_{M}. In panels A and B, parameter values are as in Fig. 1B, except $\alpha = 184$ bacteria/mM and $v = 0.514$ cm/h. Discretization parameters are $\delta x = 0.02$ cm, $\delta t = 2 \times 10^{-4}$ h. The bin size used is 0.5 cm. **C** and **D:** Corresponding numerical solutions of the deterministic system. In panels C and D, parameter values are as in Fig. 1B, except $v = 0.514$ cm/h. For direct comparison with the stochastic results shown in panels A and B, curves have been rescaled, the black one by $\alpha_d/(M_{0,d}\alpha_s)$, the pink one by α_s/α_d, and the purple one by $1/M_{0,d}$, where α_d is the value of α used in the deterministic case (see Fig. 1B), while α_s is the one employed in the stochastic case, and $M_{0,d}$ is the initial local mutant concentration at x_{M} in the deterministic description.

One of the main results of our study is that the fixation probability of a mutant is given by $1/N_{\mathrm{A}}$ instead of $1/N_{\mathrm{T}}$. Fig. S17 demonstrates that this result is validated by our numerical simulations.

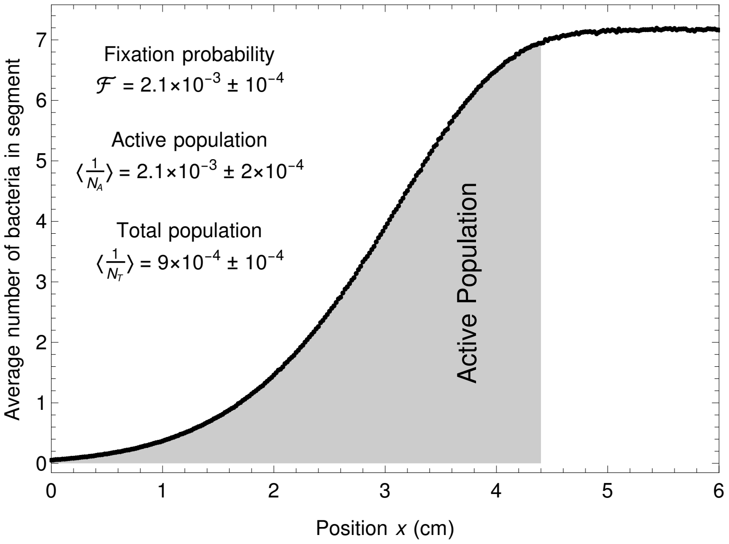

Figure S17: Fixation probability is equal to the inverse active population size. The curve shows the average profile of the bacterial population in the stochastic simulations run in panels A and B of Fig. S16. The total population size is proportional to the total area below this curve, while the active population size (defined in section S9, see also Fig. 4 of main text) is proportional to the area shaded in gray. The fixation probability $\mathcal{F}$ is computed from our stochastic simulation results as $\mathcal{F} = N_F/N_R$, where N_F is the number of fixation events and N_R the number of runs. The associated confidence interval is given by $(N_F \pm \sqrt{N_F})/N_R$. The inverses of the total and of the active population size are also given, averaged over realizations and over time. The associated confidence intervals are estimated via standard deviations.

Furthermore, Fig. S18A-B shows the distribution of mutant extinction times and mutant fixation times. Most extinction times are relatively short. The peak of the distribution of the position of mutant apparition (pink curve of panel A of Fig. S16) is about 2 cm from the downstream boundary, which takes about 4 h to cross at the velocity v employed here. Consistently, this is also the peak time for extinction events. Fixation times are much longer. Since the effective population size is the active population size, of the order of 470 and the replication rate is $r = 0.42$ h^{-1}, the fixation time is expected to be of the order of $N_{\rm A}/r \simeq 1000$ h, which is indeed the right order of magnitude. As bacterial populations in the gut are very large, fixation times will accordingly be very large. Fig. S18C shows the average proportion of mutants versus time. At the time of mutant apparition, it is the inverse of the total population size, but very quickly this mutant proportion becomes close to the inverse of the active population size. Therefore, the effect of active versus total population size matters much earlier than the fixation time.

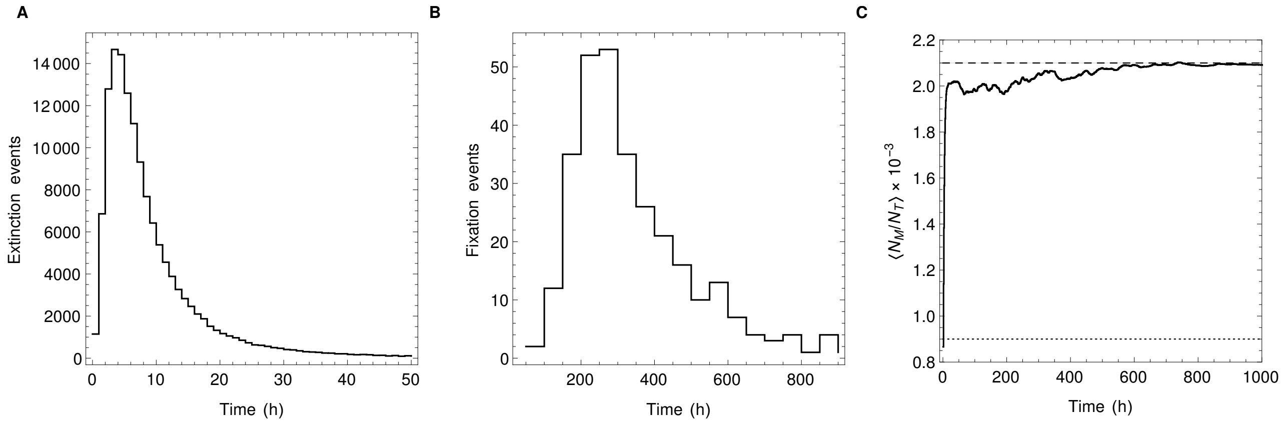

Figure S18: Time scales in stochastic simulations. A: Histogram of mutant extinction times. **B:** Histogram of mutant fixation times. **C:** Curve: average proportion of mutants versus time after mutant apparition. Dashed line: $\langle 1/N_{\rm A} \rangle$. Dotted line: $\langle 1/N_{\rm T} \rangle$. Upon mutant apparition, as there is one mutant, the mutant proportion is $1/N_{\rm T}$. In the long term, as the population will be either all wild-type or all mutant, the average mutant proportion tends to the fixation probability. But the time scale over which the mutant proportion becomes close to $1/N_{\rm A}$ is small compared to the time to fixation.

References

[1] J. Crank. *The Mathematics of Diffusion*. Oxford science publications. Clarendon Press, 1979.

[2] D. Labavić, C. Loverdo, and A.-F. Bitbol. Hydrodynamic flow and concentration gradients in the gut enhance neutral bacterial diversity, (Version v1.0.0), http://doi.org/10.5281/zenodo.4704653. Version v1.0.0. *Zenodo*, 2021.

[3] J. Cremer, I. Segota, C. Y. Yang, M. Arnoldini, J. T. Sauls, Z. Zhang, E. Gutierrez, A. Groisman, and T. Hwa. Effect of flow and peristaltic mixing on bacterial growth in a gut-like channel. *Proc. Natl. Acad. Sci. USA*, 113(41):11414–11419, 2016.

[4] R. A. Fisher. The wave of advance of advantageous genes. *Ann. Eugenics*, 7:353–369, 1937.

[5] P. Fife. *Mathematical aspects of reacting and diffusing systems*. Springer-Verlag, 1979.

[6] J. D. Murray. *Mathematical Biology I – An Introduction*. Springer-Verlag, 1989.

[7] O. Hallatschek, P. Hersen, S. Ramanathan, and D. R. Nelson. Genetic drift at expanding frontiers promotes gene segregation. *Proc. Natl. Acad. Sci. USA*, 104:19926–19930, 2007.

[8] O. Hallatschek and D. R. Nelson. Gene surfing in expanding populations. *Theor Popul Biol*, 73(1):158–170, 2008.

[9] J. M. Travis, T. Munkemuller, O. J. Burton, A. Best, C. Dytham, and K. Johst. Deleterious mutations can surf to high densities on the wave front of an expanding population. *Mol. Biol. Evol.*, 24(10):2334–2343, 2007.

[10] K. S. Korolev, M. Avlund, O. Hallatschek, and D. R. Nelson. Genetic demixing and evolution in linear stepping stone models. *Rev Mod Phys*, 82(2):1691–1718, 2010.

[11] L. Bosshard, I. Dupanloup, O. Tenaillon, R. Bruggmann, M. Ackermann, S. Peischl, and L. Excoffier. Accumulation of Deleterious Mutations During Bacterial Range Expansions. *Genetics*, 207(2):669–684, October 2017.

[12] M. Gralka, F. Stiewe, F. Farrell, W. Mobius, B. Waclaw, and O. Hallatschek. Allele surfing promotes microbial adaptation from standing variation. *Ecol. Lett.*, 19(8):889–898, August 2016.

[13] G. Birzu, O. Hallatschek, and K. S. Korolev. Fluctuations uncover a distinct class of traveling waves. *Proc Natl Acad Sci U S A*, 115(16):E3645–E3654, April 2018.

[14] A. Kolmogorov, I. Petrovsky, and N. Piscounoff. Etude de l'équation de la diffusion avec croissance de la quantite de matiere et son application à un probleme biologique. *Moscow University, Bull. Math.*, 1:1–25, 1937.

[15] B. Perthame. *Parabolic Equations in Biology – Growth, Reaction, Movement and Diffusion*. Springer, 2015.

[16] M. D. Bramson. Convergence of solutions of the Kolmogorov equation to travelling waves. *Mem. Am. Math. Soc.*, 44(285):1–190, 1983.

[17] E. Brunet. Some aspects of the Fisher-KPP equation and the branching Brownian motion, 2016.

[18] J. Cremer, M. Arnoldini, and T. Hwa. Effect of water flow and chemical environment on microbiota growth and composition in the human colon. *Proc. Natl. Acad. Sci. USA*, 114(25):6438–6443, 2017.

[19] C Rose, A. Parker, B. Jefferson, and E. Cartmell. The characterization of feces and urine: a review of the literature to inform advanced treatment technology. *Critical reviews in environmental science and technology*, 45(17):1827–1879, 2015.

[20] N. McNeil. The contribution of the large intestine to energy supplies in man. *The American journal of clinical nutrition*, 39(2):338–342, 1984.

[21] J. Debongnie and S. Phillips. Capacity of the human colon to absorb fluid. *Gastroenterology*, 74(4):698–703, 1978.

[22] K. Kovárová-Kovar and T. Egli. Growth kinetics of suspended microbial cells: from single-substrate-controlled growth to mixed-substrate kinetics. *Microbiology and molecular biology reviews*, 62(3):646–666, 1998.

[23] I. Ramirez, E. I. Volcke, R. Rajinikanth, and J.-P. Steyer. Modeling microbial diversity in anaerobic digestion through an extended adm1 model. *Water research*, 43(11):2787–2800, 2009.

[24] A. W. Lawrence and P. L. McCarty. Kinetics of methane fermentation in anaerobic treatment. *Journal (Water Pollution Control Federation)*:R1–R17, 1969.

[25] S. L. Gorbach, A. G. Plaut, L. Nahas, L. Weinstein, G. Spanknebel, and R. Levitan. Studies of intestinal microflora: II. microorganisms of the small intestine and their relations to oral and fecal flora. *Gastroenterology*, 53(6):856–867, 1967.

[26] Biomechanics of the digestive system. In T. Yamaguchi, T. Ishikawa, and Y. Imai, editors, *Integrated Nano-Biomechanics*, Micro and Nano Technologies, pages 71–99. Elsevier, Boston, 2018.

[27] J. Cummings, D. Jenkins, and H. Wiggins. Measurement of the mean transit time of dietary residue through the human gut. *Gut*, 17(3):210–218, 1976.

[28] M. Proano, M. Camilleri, S. F. Phillips, G. M. Thomforde, M. L. Brown, and R. L. Tucker. Unprepared human colon does not discriminate between solids and liquids. *American Journal of Physiology-Gastrointestinal and Liver Physiology*, 260(1):G13–G16, 1991.

[29] R. Sender, S. Fuchs, and R. Milo. Revised estimates for the number of human and bacteria cells in the body. *PLoS Biology*, 14(8):e1002533, 2016.

[30] B. Gibson, D. J. Wilson, E. Feil, and A. Eyre-Walker. The distribution of bacterial doubling times in the wild. *Proceedings of the Royal Society B*, 285(1880):20180789, 2018.

[31] W. J. Ewens. *Mathematical Population Genetics.* Springer-Verlag, 1979.

[32] O. M. Ghosh and B. H. Good. Emergent evolutionary forces in spatial models of luminal growth in the human gut microbiota. *bioRxiv*, 2021.

[33] M. J. Claesson, O. O'Sullivan, Q. Wang, J. Nikkilä, J. R. Marchesi, H. Smidt, W. M. De Vos, R. P. Ross, and P. W. O'Toole. Comparative analysis of pyrosequencing and a phylogenetic microarray for exploring microbial community structures in the human distal intestine. *PloS one*, 4(8):e6669, 2009.

[34] T. Bataillon and S. F. Bailey. Effects of new mutations on fitness: insights from models and data. *Ann N Y Acad Sci*, 1320:76–92, 2014.

[35] S. F. Levy, J. R. Blundell, S. Venkataram, D. A. Petrov, D. S. Fisher, and G. Sherlock. Quantitative evolutionary dynamics using high-resolution lineage tracking. *Nature*, 519(7542):181–186, 2015.

[36] L. Robert, J. Ollion, J. Robert, X. Song, I. Matic, and M. Elez. Mutation dynamics and fitness effects followed in single cells. *Science*, 359(6381):1283–1286, 2018.
